# Cell-Autonomous Progeroid Changes in Conditional Mouse Models for Repair Endonuclease XPG Deficiency

**DOI:** 10.1371/journal.pgen.1004686

**Published:** 2014-10-09

**Authors:** Sander Barnhoorn, Lieneke M. Uittenboogaard, Dick Jaarsma, Wilbert P. Vermeij, Maria Tresini, Michael Weymaere, Hervé Menoni, Renata M. C. Brandt, Monique C. de Waard, Sander M. Botter, Altaf H. Sarker, Nicolaas G. J. Jaspers, Gijsbertus T. J. van der Horst, Priscilla K. Cooper, Jan H. J. Hoeijmakers, Ingrid van der Pluijm

**Affiliations:** 1 Department of Genetics, Erasmus University Medical Center, Rotterdam, The Netherlands; 2 Department of Neuroscience, Erasmus University Medical Center, Rotterdam, The Netherlands; 3 Department of Intensive Care, VU University Medical Center, Amsterdam, The Netherlands; 4 Uniklinik Balgrist, Zürich, Switzerland; 5 Life Sciences Division, Lawrence Berkeley National Laboratory, Berkeley, California, United States of America; 6 Department of Vascular Surgery, Erasmus University Medical Center, Rotterdam, The Netherlands; The Scripps Research Institute, United States of America

## Abstract

As part of the Nucleotide Excision Repair (NER) process, the endonuclease XPG is involved in repair of helix-distorting DNA lesions, but the protein has also been implicated in several other DNA repair systems, complicating genotype-phenotype relationship in XPG patients. Defects in XPG can cause either the cancer-prone condition xeroderma pigmentosum (XP) alone, or XP combined with the severe neurodevelopmental disorder Cockayne Syndrome (CS), or the infantile lethal cerebro-oculo-facio-skeletal (COFS) syndrome, characterized by dramatic growth failure, progressive neurodevelopmental abnormalities and greatly reduced life expectancy. Here, we present a novel (conditional) *Xpg^−/−^* mouse model which -in a C57BL6/FVB F1 hybrid genetic background- displays many progeroid features, including cessation of growth, loss of subcutaneous fat, kyphosis, osteoporosis, retinal photoreceptor loss, liver aging, extensive neurodegeneration, and a short lifespan of 4–5 months. We show that deletion of XPG specifically in the liver reproduces the progeroid features in the liver, yet abolishes the effect on growth or lifespan. In addition, specific XPG deletion in neurons and glia of the forebrain creates a progressive neurodegenerative phenotype that shows many characteristics of human XPG deficiency. Our findings therefore exclude that both the liver as well as the neurological phenotype are a secondary consequence of derailment in other cell types, organs or tissues (e.g. vascular abnormalities) and support a cell-autonomous origin caused by the DNA repair defect itself. In addition they allow the dissection of the complex aging process in tissue- and cell-type-specific components. Moreover, our data highlight the critical importance of genetic background in mouse aging studies, establish the *Xpg^−/−^* mouse as a valid model for the severe form of human XPG patients and segmental accelerated aging, and strengthen the link between DNA damage and aging.

## Author Summary

Accumulation of DNA damage has been implicated in aging. Many premature aging syndromes are due to defective DNA repair systems. The endonuclease XPG is involved in repair of helix-distorting DNA lesions, and XPG defects cause the cancer-prone condition xeroderma pigmentosum (XP) alone or combined with the severe neurodevelopmental progeroid disorder Cockayne syndrome (CS). Here, we present a novel (conditional) *Xpg^−/−^* mouse model which -in a C57BL6/FVB F1 hybrid background- displays many progressive progeroid features, including early cessation of growth, cachexia, kyphosis, osteoporosis, neurodegeneration, liver aging, retinal degeneration, and reduced lifespan. In a constitutive mutant with a complex phenotype it is difficult to dissect cause and consequence. We have therefore generated liver- and forebrain-specific *Xpg* mutants and demonstrate that they exhibit progressive anisokaryosis and neurodegeneration, respectively, indicating that a cell-intrinsic repair defect in neurons can account for neuronal degeneration. These findings strengthen the link between DNA damage and the complex process of aging.

## Introduction

If DNA damage, either inflicted from exogenous or endogenous sources, cannot be repaired, this has detrimental consequences for an organism ranging from transcription blocks, permanent cell cycle arrest and mutations, to cell death. In the end, this unrepaired DNA damage contributes to the onset and progression of the aging process, as well as to cancer [1]–[3]. Cells are equipped with a set of elaborate DNA repair mechanisms integrated into a complex DNA damage response machinery that jointly attempt to fix the unrepaired DNA [4]. One such DNA repair mechanism is the Nucleotide Excision Repair (NER) pathway that removes a wide category of helix-distorting lesions, such as those induced by UV and bulky chemical adducts, in a tightly coordinated process involving over 30 proteins [5]–[7]. NER can be divided into two subpathways based on the mode of damage recognition. The Global Genome (GG-)NER subpathway specifically involves the XPC and XPE protein complexes, and probes the entire genome for lesions that disrupt base-pairing [5], [7]–[9]. Transcription-Coupled (TC-)NER, on the other hand, detects helix-distorting lesions that stall transcription in the transcribed strand of expressed genes, and hence enables resumption of transcription. TC-NER is independent of XPC and XPE and specifically involves proteins such as CSA, CSB and UVSSA [8], [10], [11]. After lesion recognition, the subsequent ‘cut-and-patch’ core repair reaction encompasses local opening of the DNA helix and lesion verification, performed by the TFIIH complex together with XPA. Both correctly position the structure-specific endonucleases ERCC1/XPF and XPG for strand-specific excision of the lesion as part of a 22–30 bp oligonucleotide [5], [7], [12]. Finally, the gap is filled by repair synthesis and closed by ligation [5], [7], [12].

Multiple NER proteins have been attributed additional roles, both in DNA repair pathways other than NER, and in transcription regulation. For instance, TFIIH is an essential component of the general transcription machinery [13], [14], but also other NER factors, including XPG and CSB, have been implicated in transcription regulation [15]–[18]. The 5′ endonuclease ERCC1/XPF participates in the repair of interstrand crosslinks [19], [20] and subpathways of DNA double-strand break repair [21]. XPC, CSB, and XPG have been individually implicated in promoting base excision repair (BER) of oxidative DNA damage [22]–[30]. TFIIH and XPG are, together with CSB, thought to be involved in the early steps of Transcription-Coupled Repair (TCR), and XPG interacts directly with both CSB and RNA Polymerase II [31]. Although still controversial, there are accumulating reports that TCR not only directs NER to blocked transcription but may also recruit BER for preferential repair of oxidative DNA damage in transcribed strands [32]–[34]. Such a mechanism might be related to the roles of both CSB and XPG in promoting BER more globally. If correct, it could explain the much greater consequences for the organism of TCR defects compared to defects in NER alone (see below) [35].

A number of rare, autosomal recessive disorders resulting from mutations in NER genes underscore the importance of genome maintenance for the prevention of cancer as well as aging [3]. NER-associated diseases are characterized by sun (UV) hypersensitivity and include xeroderma pigmentosum (XP), UV-sensitivity syndrome (UVSS), Cockayne syndrome (CS), cerebro-oculo-facio-skeletal (COFS) syndrome, XPF-ERCC1 (XFE) progeroid syndrome, trichothiodystrophy (TTD) and disorders that combine the symptoms of these syndromes, including XP/CS [35]–[39]. XP originates from defects in GG-NER or total NER activity and is characterized by an over 2000-fold increased risk of cancer in sun-exposed skin and, to a much lesser extent, in internal organs [36]. XP patients may also develop progressive neurological symptoms and neuronal degeneration depending on the severity of the total NER deficiency [36], [39], [40]. UVSS is characterized by skin UV hypersensitivity without actually developing skin cancer. UVSS results from the selective loss of TC-NER function as a consequence of mutations in the proteins involved in detection of UV-induced transcription-blocking DNA lesions, i.e. UVSSA, CSA, and CSB [11], [35], [41]–[44]. Mutations in CSA and CSB, however, generally cause CS, a heterogeneous multisystem disorder that, in addition to UV-sensitivity, is characterized by severe growth failure and cachexia, accelerated aging features, short lifespan, and progressive sensori-neuronal abnormalities [38], [45]. The severe symptoms of CS cannot be explained by the loss of TC-NER function as they do not occur in fully NER-deficient XP patients and TC-NER deficient UVSS patients. Therefore, CS symptoms have been linked to additional, yet incompletely, defined functions of CSA and CSB in DNA repair, transcription regulation, other processes, or a combination of deficiencies [3], [46], [47]. The same applies for mutations in the down-stream NER factors XPB, XPD, XPF, ERCC1 and XPG that cause combined XP/CS, or severe developmental/degenerative multisystem disorders such as COFS and XFE that share multiple features with severe CS forms [35], [48]–[51]. Thus CS symptoms can result from mutations in multiple proteins that operate together in NER, but the symptoms caused by these mutations cannot be explained by NER deficiency alone, raising questions about the identities of these non-NER activities underlying CS symptoms and the extent to which different symptoms reflect deficits of different cellular processes [35], [46].

Mutations in the structure-specific NER 3′-endonuclease XPG are rare, with less than 20 patients and 25 mutant alleles described so far [52]–[55], and cause a spectrum of disease phenotypes varying from XP to XP/CS and COFS [53]. Point mutations that selectively eliminate XPG nuclease activity cause XP, while C-terminal truncations, destabilizing point mutations, and mutations that abolish the interaction between XPG and the basal transcription factor TFIIH cause XP/CS and COFS [52]–[58]. These data support the notion that a deficient function of XPG outside NER is responsible for the severe CS symptoms [15], [52], [53], [55]–[57].

For most NER disorders, mouse mutants have been generated that mimic the genetic defect found in patients, and to various extents reproduce XP and CS-like features as well as the progeroid hallmarks found in the corresponding human syndrome [59]–[62]. Accordingly, *Xpg*-null (*Xpg^−/−^*) mice were found to develop a severe phenotype characterized by growth deficiency and very short lifespan, resembling severe XP/CS [63]. In contrast, *Xpg* mutant mice carrying amino acid substitutions that selectively abolish the nuclease function of XPG (*Xpg^E791A^* and *Xpg^D811A^*) show severe UV-sensitivity but normal lifespan, hence, reproducing the XP phenotype [64], [65]. In addition, a mutant XPG construct containing a C-terminal truncation lacking the last 360 amino acids that was made to mimic the genotype of some XP-G/CS patients, developed a growth deficiency and short-living phenotype resembling that of *Xpg^−/−^* mice, albeit somewhat milder [65]. Yet another C-terminal truncation mutant lacking the last 180 amino acids showed a normal lifespan, but produced a CS-like growth-deficient short-living phenotype after crossing with *Xpa^−/−^* mice that are already fully NER-deficient [65], [66]. Significantly, the same conversion of a normal lifespan into a short-living mouse model is observed after crossing CSA- or CSB-deficient CS mice with total NER- (*Xpa^−/−^*) or GG-NER (*Xpc^−/−^*) deficient mouse models [67]–, but not by crossing NER-deficient *Xpg^D811A^* with *Xpa^−/−^* mice [66]. Together these data indicate a deleterious synergistic interaction between NER deficiency and loss of non-NER activities that underlie CS. Furthermore, they show that the C-terminus of XPG could play a role in the CS symptoms, and that the XPG-deficient *Xpg^−/−^* mice may reproduce the phenotype of *Xpa^−/−^Csb^−/−^*, *Xpc^−/−^Csb^−/−^* or *Xpa^−/−^Csa^−/−^* double mutant mice [62], [66]–[69].

In previous analyses we clearly observed progeroid characteristics in many NER mutant mouse models including *Xpa/Csb*, *Xpb*, *Xpd*, and *Ercc1* mutants [51],[68],[70]–[72], yet the occurrence of progeroid features in *Xpg^−/−^* mice has hitherto been poorly established, mostly due to their very short lifespan. Since we are particularly interested in the effect of *Xpg* deletion on organ-specific aging, we generated a conditional *Xpg* mutant. As genetic background can have a significant effect on phenotype development, we first re-examined the pathological characteristics of *Xpg^−/−^* mice in a C57BL6/FVB F1 hybrid background, as was previously described for *Ercc1* mutant mice [51], [72]. In this hybrid background *Xpg^−/−^* mice lived longer and presented progeroid features including cachexia and osteoporosis with pronounced degenerative phenotypes in both liver and brain. We next studied the effect of liver- and forebrain-specific inactivation of *Xpg*, showing that the observed phenotypes are indeed due to lack of XPG protein. Together our data show that, consistent with previous data in ERCC1- and CSB/XPA-deficient mice, *Xpg^−/−^* mice develop a multisystem progeroid degenerative phenotype.

## Results

### Generation of NER-deficient *Xpg^−/−^* mice

**Figure 1:**
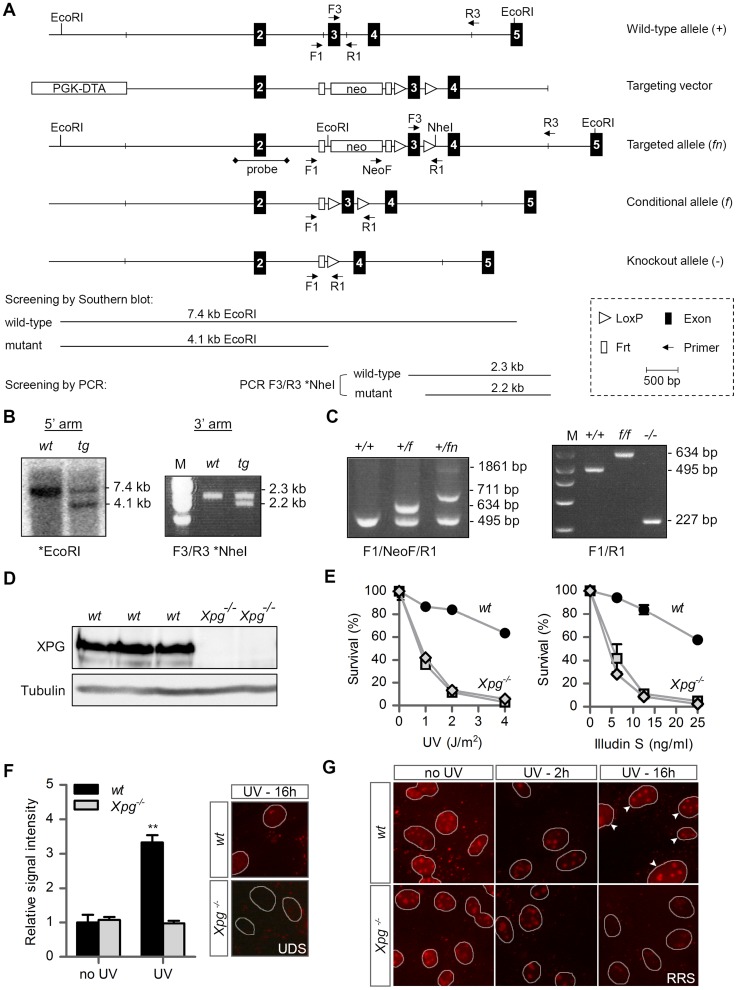
Generation of *Xpg^−/−^* mice. (A) Genomic organization and disruption strategy for *Xpg* depicting the wild type allele (*+*), the targeting construct, the targeted allele (*fn*), the conditional allele after Flp-mediated recombination of *Frt* sites (*f*) and the targeted *Xpg* allele following subsequent Cre-mediated recombination of *LoxP* sites (*−*). Exons 2–5 are indicated by black boxes. PCR primers are shown as arrows. (B) Southern blot and PCR analysis of an ES clone showing the correct insertion of the targeting construct. ES cell genomic DNA was digested with EcoRI for Southern blot analysis and hybridized with a 0.9 kb DpnI probe. The wild type (*wt*) allele yields a 7.4-kb fragment whereas the targeted (*tg*) allele yields a 4.1-kb fragment. The NheI-digested PCR product shows the 2.3-kb and 2.2-kb bands corresponding with the *wt* and *tg* allele, respectively (see also panel A). (C) PCR detection of mouse genotypes using the primers F1, NeoF and R1 as indicated as in A. (D) Immunoblot analysis of extracts from *Xpg^−/−^* and *wt* MDFs using a rabbit polyclonal antibody raised against a peptide conserved between human and mouse XPG. Tubulin is used as loading control. (E) Primary *Xpg^−/−^* and *wt* MDFs, cultured at low (3%) O_2_ levels were irradiated with the indicated doses of UV-C (left) or treated with the indicated doses of Illudin S for 1 h (right). After 48 h recovery, survival was assessed by cell count. (F) UV-induced UDS in primary *Xpg^−/−^* and *wt* MDFs reveals a severe GG-NER defect in *Xpg^−/−^* cells. MDFs were irradiated with 16 J/m^2^ of UV-C. UDS levels are expressed relative to the non-irradiated *wt* cells. (G) UV-induced RRS in primary *Xpg^−/−^* and *wt* MDFs reveals a severe TC-NER defect in *Xpg^−/−^* cells. MDFs were irradiated with 16 J/m^2^ of UV-C. 16 h after UV irradiation the *wt* cells show recovery of RNA synthesis, while *Xpg^−/−^* MDFs only show residual activity in nucleoli (rRNA transcription). Arrowheads indicate nuclei. Error bars indicate standard error of the mean. **p<0.01.

To generate a Cre-inducible *Xpg* knockout allele we flanked the third exon of *Xpg* with *LoxP* sites (Figure 1A). Deletion of this exon causes a frame shift and a premature translational stop immediately after exon 2 at amino acid residue 89 (instead of the full-length 1170). After transfection to 129 ES cells and selection of properly targeted clones (Figure 1B and Materials and Methods), two independent transfected clones were used to generate germ-line transmitting chimeras (Figure 1C). Heterozygous males, carrying the conditional *Xpg* allele, were crossed to females ubiquitously expressing *Flp* for excision of the Neomycin cassette and to yield mice that are heterozygous for the floxed *Xpg* (*Xpg^f^*) allele. *Xpg^f^* ^/+^ mice were backcrossed and maintained in FVB/N background. To generate *Xpg* mice carrying a knockout allele (*Xpg^−^*, Figure 1A), *Xpg^f^* ^/+^ mice were crossed to *Cag-Cre* mice, which ubiquitously express Cre recombinase from germline [73], yielding heterozygous *Xpg^+/−^* animals (Figure 1C). *Xpg^+/−^* animals were>10 times backcrossed into C57BL6 or FVB/N genetic backgrounds. Unless otherwise stated, experiments were performed with *Xpg^−/−^* mice in the C57BL6/FVB F1 hybrid background obtained from intercrossing C57BL6 *Xpg^+/−^*×FVB/N *Xpg^+/−^* animals to minimalize background specific effects (see below).

The presence of a premature stop codon in the *Xpg^−^* allele was confirmed by sequencing *Xpg* cDNA from liver of *Xpg^−/−^* mice (Figure S1A). Accordingly, Western immunoblot analysis with an antibody raised against the central spacer region (R-domain) of XPG shows the absence of XPG protein product in mouse dermal fibroblasts (MDFs) isolated from *Xpg^−/−^* mice (Figure 1D). Next, we tested DNA repair deficiency of *Xpg^−/−^* MDFs. In accordance with complete NER deficiency, *Xpg^−/−^* MDFs showed an almost 10-fold hypersensitivity to UV, similar to fully NER-defective MDFs derived from *Xpa^−/−^* mice (Figure 1E) [74]. In addition, *Xpg^−/−^* MDFs were hypersensitive to treatment with Illudin S (Figure 1E), consistent with the loss of TC-NER function [75], and were deficient in UV-induced unscheduled DNA synthesis in line with loss of GG-NER activity (Figure 1F). Also, recovery of RNA synthesis after UV exposure was almost completely abolished in *Xpg^−/−^* MDFs, further demonstrating loss of TC-NER activity (Figure 1G). *Xpg^−/−^* MDFs showed no increased sensitivity to potassium bromate (KBrO_3_) which causes oxidative DNA lesions, and a minimal increased sensitivity to the cross-linking agent cisplatin (Figure S1B).

### Genetic background affects perinatal viability and lifespan of *Xpg^−/−^* mice

**Figure 2:**
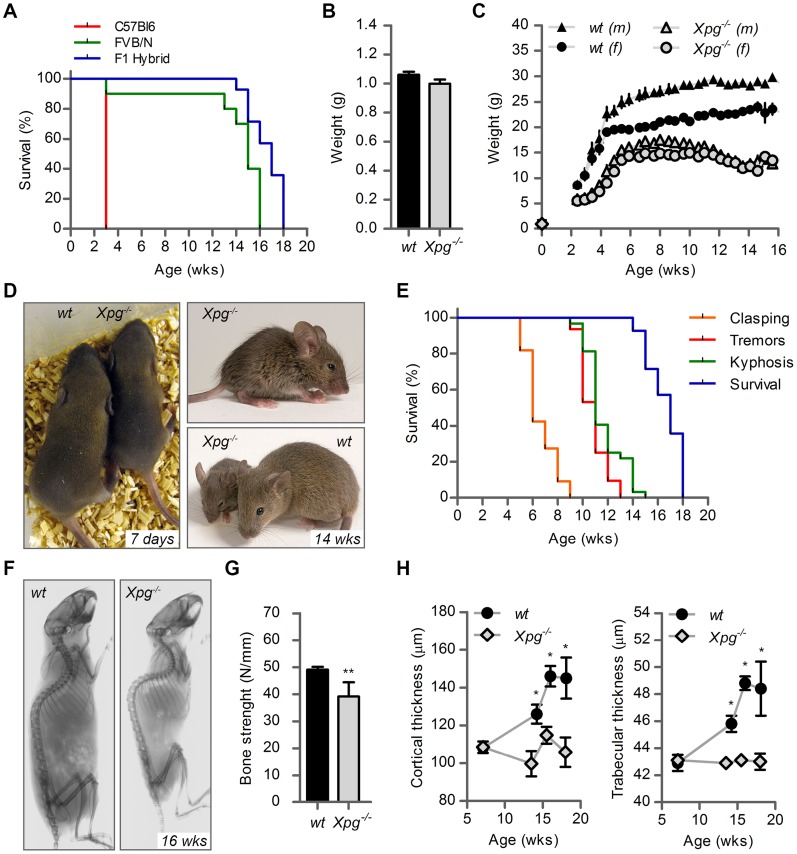
Progeroid characteristics of *Xpg^−/−^* mice. (A) Survival of *Xpg^−/−^* mice in a C57Bl6 (red), FVB/N (green) or C57Bl6/FVB F1 hybrid (blue) background; n = 5 (C57Bl6), n = 10 (FVB/N), n = 14 (C57Bl6/FVB F1 hybrid). (B) Average body weight of embryonic 17.5-day old F1 hybrid *Xpg^−/−^* and wild type (*wt*) littermates; n≥12 animals/group. (C) Average body weight of F1 hybrid *wt* males (black triangles), *wt* females (black circles), *Xpg^−/−^* males (grey triangles), and *Xpg^−/−^* females (grey circles); n≥4 animals/group. (D) Left: Photograph of a 7-day old F1 hybrid *Xpg^−/−^* and *wt* littermate, showing no apparent differences except a slightly smaller size. Top right: Photograph of a 14-week old *Xpg^−/−^* mouse. Bottom right: Side by side comparison of the same 14-week old *Xpg^−/−^* and *wt* littermate showing a pronounced growth deficiency of the *Xpg^−/−^* mouse. (E) Onset of hind limb clasping (orange), tremor (red) and kyphosis (green) with age and survival of F1 hybrid *Xpg^−/−^* mice; n = 33 (clasping, tremor and kyphosis), n = 14 (survival). (F) CT-scan of a 16-week old F1 hybrid *wt* (left) and *Xpg^−/−^* (right) mouse showing prominent curvature of the spine (kyphosis) in the *Xpg^−/−^* mouse. (G) Bone strength of F1 hybrid *Xpg^−/−^* and *wt* mice analyzed by a 3-point-bending assay of the femur at an average age of 15 weeks; n≥6 animals/group. (H) Cortical (left) and trabecular (right) thickness of the femora of F1 hybrid *Xpg^−/−^* and *wt* mice at different ages; n = 4 animals/group. Error bars indicate standard error of the mean. *p<0.05, **p<0.01.

In view of a significant effect of genetic background on embryonic lethality and lifespan in ERCC1-deficient mice [51], [72], we examined whether a similar genetic background effect occurred in *Xpg* ^−/−^ mice, by comparing birth frequencies and lifespan of *Xpg^−/−^* mice in a C57BL6, FVB/N or a C57BL6/FVB F1 hybrid background. In C57BL6 background birth frequencies were below Mendelian expectations (∼8%, Table 1), whereas in the FVB/N and C57BL6/FVB F1 hybrid background birth frequencies were Mendelian and near-Mendelian, respectively (Table 1). Also the lifespan of *Xpg^−/−^* animals was strongly dependent on genetic background, with C57BL6 *Xpg^−/−^* mice showing a lifespan of 3 weeks, and *Xpg^−/−^* animals in FVB/N and C57BL6/FVB F1 hybrid background living for 15–18 weeks (Figure 2A).

**Table 1:** *Xpg^−/−^* mice are born below Mendelian ratio in a C57BL6 background.

Total	+/+	+/−	−/−	Genetic background
**60**	22 (36.7%)	33 (55.0%)	5 (8.3%)**	C57Bl6
**39**	11 (28.2%)	16 (41.0%)	12 (30.8%)	FVB/N
**545**	156 (28.6%)	276 (50.6%)	112 (20.6%)*	F1 (C57Bl6/FVB)

Ratio of knockout mice born in different genetic backgrounds.

*p<0.05;

**p<0.01 deviation from Mendelian ratio (ChiSquaredTest).

### Cachexia, short lifespan and osteoporosis in *Xpg^−/−^* mice

Further analysis of C57BL6/FVB F1 *Xpg^−/−^* mice showed that they had the same size and weight as wild type and heterozygote littermates at late embryonic stage (E17.5; Figure 2B and C). However, after birth, *Xpg^−/−^* mice showed reduced growth and weight gain compared to controls, and stopped growing at 6–8 weeks when their body weight was about 65–70% of that of wild type littermates (Figure 2C). From 10–11 weeks, body weights declined and the *Xpg^−/−^* mice became progressively cachectic (Figure 2C and D). At 14 weeks all *Xpg^−/−^* mice were severely runted (Figure 2D), and the mice died a few weeks thereafter between 15–18 weeks of age (Figure 2A and E). The growth deficiency was paralleled by the development of kyphosis (Figure 2D and F). In addition, *Xpg^−/−^* mice progressively developed neurological symptoms, including clasping of the hind-limbs when lifted by their tails (Figure S2A), and at a later time point fine tremors (Figure 2E). Accelerating rotarod and grip strength tests in 14-week old *Xpg^−/−^* mice revealed severe motor deficits and muscle weakness at this age (Figure S2B and C).

Computed tomography (CT) confirmed severe kyphosis in *Xpg^−/−^* mice at 16 weeks of age (Figure 2F). To further examine skeletal abnormalities and the occurrence of osteoporosis as observed in other NER-deficient mouse models [68], [76]–[79], we measured several bone parameters using femoral bones. Analysis of bone strength revealed decreased strength of the *Xpg^−/−^* femoral bone at 14–16 weeks (Figure 2G). Micro-CT analysis showed that the thickness of the trabeculae and cortex of the femoral bones was significantly smaller in *Xpg^−/−^* compared to wild type mice at 14–17 weeks, but not yet at 7 weeks (Figure 2H). Overall, these data indicate a progressive increase of age-related features such as osteoporosis.

### Mild progeroid features and a ‘survival-like’ stress response in livers of *Xpg^−/−^* mice

**Figure 3:**
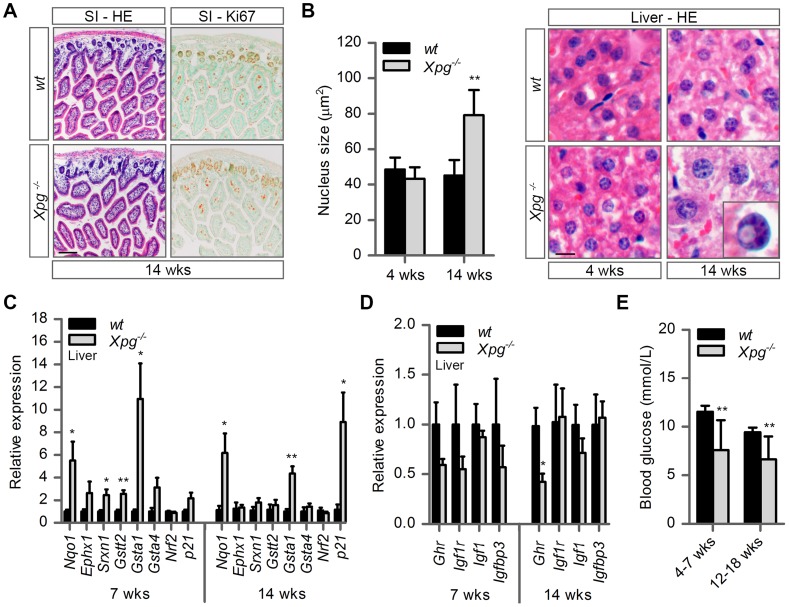
Intestine and liver phenotype of *Xpg^−/−^* mice. (A) Representative images of HE and Ki67 stained small intestine (SI) of 14-week old *Xpg^−/−^* and wild type (*wt*) mice showing no gross morphological differences. (B) Average nucleus size of hepatocytes in the liver of 4- and 14-week old *Xpg^−/−^* and *wt* mice; n≥3 animals/group. Bottom right: magnification of a nuclear inclusion found sporadically in liver sections of 14-week old *Xpg^−/−^* mice. (C) Relative mRNA expression levels of several antioxidant genes and the DNA damage response gene *p21* in liver tissue of 7- and 14-week old *Xpg^−/−^* and *wt* mice. All values are corrected for *TubG2, Hprt, and Rps9* (Table S1) expression as internal standard and normalized to the 7-week old *wt* expression levels; n = 4 animals/group. (D) Relative expression levels of the somatotrophic genes *Ghr*, *Igf1r*, *Igf1*, and *Igfbp3* in liver tissue of 7- and 14-week old *Xpg^−/−^* and *wt* mice. All values are corrected for *TubG2, Hprt, and Rps9* expression and normalized to the 7-week old *wt* expression levels; n = 4 animals/group. (E) Average basal blood glucose levels in groups of 4–7 and 12–18 week old *Xpg^−/−^* and *wt* mice; n≥15 animals/group. Scale bars: 50 µm (A), 10 µm (B). Error bars indicate standard error of the mean. *p<0.05, **p<0.01.

Weight loss and reduced size of *Xpg^−/−^* mice was associated with reduced weight of internal organs (Figure S3A) and with a strong reduction in the amount of subcutaneous fat (Figure S3B). The *Xpg^−/−^* mice previously reported by Harada et al. [63] showed developmental abnormalities of the gastro-intestinal tract. These gastro-intestinal abnormalities were proposed to be a major contributor of the post-natal growth failure and short lifespan (<3 weeks) of their animals [63]. However, in contrast to their data, the gastro-intestinal tract of our *Xpg^−/−^* mice had a normal size and macroscopic appearance, and showed a normal histological appearance in HE-stained sections (Figure 3A). Furthermore, staining for the proliferative cell marker Ki-67 indicated that the number of proliferative cells in the intestinal epithelium was similar between *Xpg^−/−^* and wild type mice (Figure 3A). In accord with normal function of the gastro-intestinal tract we found that food intake per gram body weight was similar between wild type and *Xpg^−/−^* animals (Figure S3C).

The liver is a central organ in many aspects of metabolic control, including regulation of circulating glucose levels and detoxification, and it plays a key role in regulation of IGF1-somatotrophic axis signaling. Previous studies have shown that ERCC1/XPF-deficient mice develop multiple liver abnormalities, in particular anisokaryosis resulting from polyploidy, and intranuclear inclusions [72], [80]–[83]. Analysis of HE-stained liver sections of our *Xpg^−/−^* mice revealed mild anisokaryosis, and increased mean nuclear size at 14 weeks, but not at 4 weeks of age (Figure 3B). In addition, sporadically, hepatocytes had intranuclear inclusions. These liver nuclear changes are a well characterized phenomenon in the aging liver, and indicate that *Xpg^−/−^* mice show features of accelerated aging in the liver similar to *Ercc1^Δ/−^* mice [72], [82].

Liver cells of ERCC1-deficient and other progeroid NER-deficient mouse mutants display changes in gene expression that encompass a downregulation of catabolic and oxidative metabolism and an upregulation of antioxidant and stress defense pathways, suggestive of a compensatory survival response to cope with increased DNA damage [51], [68], [84], [85]. To determine whether *Xpg^−/−^* liver cells also display a ‘survival-like’ stress response, we determined expression levels of selected antioxidant and somatotrophic genes by real-time PCR. Indeed, mRNA levels from a subset of antioxidant effector genes, including *Nqo1*, *Srxn1*, *Gstt2* and *Gsta1*, were significantly increased in liver homogenates of young (7-week old) *Xpg^−/−^* animals compared to controls. At 14 weeks of age, we observed a similar significant increased expression of *Nqo1* and *Gsta1* while mRNA from the other antioxidant effector genes tested showed unaltered expression (Figure 3C). Expression levels of *Nrf2*, which is a potent inducer of the antioxidant response element (ARE), were unaltered, in line with the fact that NRF2-activation is largely achieved by post-translational mechanisms [86]. As increased expression of antioxidant genes could be an indication of increased genotoxic stress, we also checked the expression of the p53-responsive kinase inhibitor *p21*, a master regulator of cell survival and death [87], which is generally increased after DNA damage and was previously shown to be elevated in livers of *Ercc1* mutant mice [88]. Expression levels of *p21* doubled at the age of 7 weeks and were massively increased at the age of 14 weeks, indicative of genotoxic stress caused by the absence of XPG. To determine changes in somatotrophic gene expression we examined mRNA levels of *Ghr, Igf1r, Igf1 and Igfbp3*. We found a two-fold suppression of *Ghr* and *Igf1r* mRNA expression at 7 weeks, and a significant downregulation of *Ghr* mRNA levels at 14 weeks of age (Figure 3D). Together the data indicate that the *Xpg^−/−^* liver in part reproduces gene expression changes observed in other short-living NER-deficient mice, which we refer to as a survival-like stress response. Finally, consistent with other NER-deficient progeroid mice [51], [85], we found significantly reduced steady-state blood glucose levels in *Xpg^−/−^* mice (Figure 3E).

### Age-related accumulation of neurodegenerative changes in *Xpg^−/−^* central nervous system

**Figure 4:**
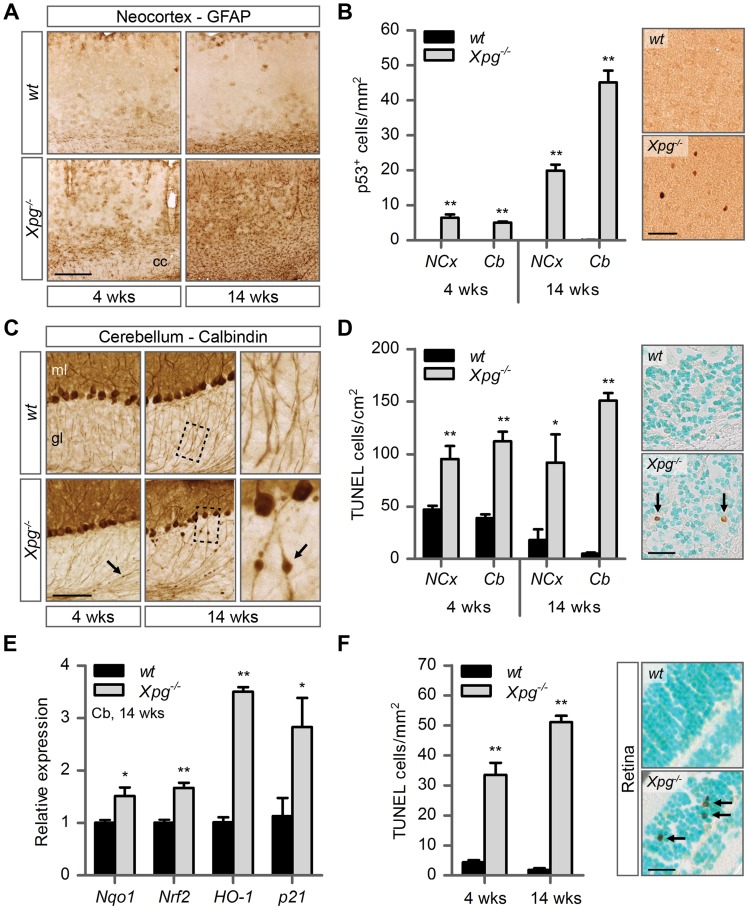
Increased cell death, degeneration and stress responses in post-mitotic tissues of *Xpg^−/−^* mice. (A) Representative images of GFAP immunostained sagittal neocortex sections of 4- and 14-week old *Xpg^−/−^* and wild type (*wt*) mice showing progressive astrocytosis in *Xpg^−/−^* mice. cc: corpus callosum. (B) Quantification of p53-positive cells per mm^2^ in neocortex (NCx) and cerebellum (Cb) sections of 4- and 14-week old *Xpg^−/−^* and *wt* mice; n = 3 (14 weeks) and the average of five sections of a 4-week old *Xpg^−/−^* and *wt* animal. (C) Representative images of calbindin immunostained sagittal cerebellum sections of 4- and 14-week old *Xpg^−/−^* and *wt* mice. Right panel: Magnification of the areas marked with dotted black boxes. Arrows indicate cerebellar torpedoes. ml: molecular layer, gl: granular layer. (D) Quantification of TUNEL-positive cells per cm^2^ in neocortex and cerebellum sections of 4- and 14-week old *Xpg^−/−^* and *wt* mice; n≥3 animals/group. Arrows indicate positive cells. (E) Relative mRNA expression levels of the antioxidant genes *Nqo1*, *Nrf2*, and *HO-1* and the DNA damage response gene *p21* in 14-week old *Xpg^−/−^* and *wt* cerebellum tissue. All values are corrected for *TubG2* expression and normalized to *wt* expression levels; n = 4 animals/group. (F) Quantification of TUNEL-positive cells per mm^2^ in retinal sections of 4- and 14-week old *Xpg^−/−^* and *wt* mice; n = 6 animals/group. Arrows indicate positive cells. Scale bars: 250 µm (A), 50 µm (B), 100 µm (C), 25 µm (D, F). Error bars indicate standard error of the mean. *p<0.05, **p<0.01.

The occurrence of neurological abnormalities and impaired motor behavior in *Xpg^−/−^* mice (Figure 2E and S2), as well as the abundant neurodegenerative features in ERCC1-deficient and combined XP/CS mouse models [89]–[91], prompted us to investigate the central nervous systems of *Xpg^−/−^* animals for neurodegenerative changes. Macroscopically, the brains and spinal cords of *Xpg^−/−^* mice showed a normal appearance, albeit somewhat smaller. In addition, the gross histological organization analyzed in thionin-stained sections appeared normal in all brain regions. As a first step to examine the occurrence of neurodegenerative changes, we examined the brains of 4- and 14-week old *Xpg^−/−^* mice immunohistologically for glial acidic filament protein (GFAP) expression, which outlines reactive astrocytosis in response to neuronal injury. A mild increase in GFAP immunostaining occurred in patches in multiple nervous system areas at 4 weeks (Figure 4A and S4A). Instead, at 14 weeks, *Xpg^−/−^* mice showed a prominent ubiquitous increase in GFAP staining throughout the entire central nervous system including spinal cord, indicative of widespread astrocytosis (Figure 4A and S4A). Double-labelling of GFAP and the microglia cell marker Iba-1 showed that the increased GFAP staining was paralleled by microglia activation, characterized by increased Iba-1 immunoreactivity and the transformation of resting microglia cells into activated cells with thicker processes and larger cell bodies (Figure S4B and C).

Next, to determine whether *Xpg^−/−^* central nervous system cells experience genotoxic stress, we studied the expression of the transcription factor p53, which is activated by multiple types of DNA damage and is expressed in neurons and macroglia of many NER-deficient mouse models including mice defective in *Ercc1*, *Csa* or *Csb* [89]–[91]. Immunohistochemistry revealed p53-positive cells in all central nervous system regions. Analysis of the p53 density in neocortex and cerebellum indicated an increase in number of p53-positive cells in brains of 14-week old compared to 4-week old *Xpg^−/−^* mice (Figure 4B). Similar to our findings in other NER mutant mice [89]–[91], double labelling of p53 with neuronal (NeuN) and astrocytic (GFAP, S100β) markers, indicated that these p53-positive cells include neurons, astrocytes (GFAP+ or S100β+; Figure S4D), and oligodendrocytes. Although not systematically investigated, we also noted that, as in other NER-deficient mice, in neocortex and cerebellar cortex the majority of p53-positive cells were neurons, while in spinal cord a large proportion of p53-positive cells were astrocytes (Figure S4E).

To obtain evidence for the occurrence of neuronal death, we analyzed calbindin staining in cerebellar cortex where it outlines Purkinje cells and enables easy detection of the degeneration of these cells [90]–[92]. Calbindin staining revealed degeneration and loss of Purkinje cells in 14-week old *Xpg^−/−^* mice (Figure 4C). Also, calbindin staining revealed sporadic Purkinje cells with abnormal dendritic morphologies and, more frequently, Purkinje cells with swellings in their proximal axon (Figure 4C). Axonal swellings (also designated torpedoes or axonal spheroids) are a common feature in neurodegenerative disorders and aging [93], that is also well documented for Purkinje cell axons of CS and XP/CS patients [94]. The presence of axonal pathology indicates that many surviving Purkinje cells in 14-week old *Xpg^−/−^* mice display compromised health. Notably, few small axonal swellings occurred in Purkinje cells axons in 4-week old *Xpg^−/−^* mice.

To further examine the extent to which neurons in *Xpg^−/−^* mice show compromised health, we examined the morphology of the Golgi apparatus in motor neurons. In a previous study in *Ercc1* mutant mice we noted that motor neurons displayed a variety of morphological abnormalities of the Golgi apparatus, and we proposed that these abnormalities reflect a heterogeneity of cellular deficits resulting from stochastic DNA damage [90]. Immunostaining for the cis-Golgi marker GM130 showed that motor neurons in *Xpg^−/−^* mice developed the same heterogeneity in morphological abnormalities of the Golgi apparatus as observed in *Ercc1^Δ/−^* mice. Double labelling of GM130 and p53 indicated that only a small subset of neurons with abnormal Golgi apparatus is p53 positive. This variability in p53 expression further illustrates the heterogeneity of degenerative events that may occur in *Xpg^−/−^* neurons (Figure S4F).

TUNEL staining to determine the amount of apoptotic cells showed a significant increase in both the cerebrum and the cerebellum at 4 as well as 14 weeks of age (Figure 4D). Finally, real-time PCR in *Xpg^−/−^* cerebellum revealed an upregulation of the p53-responsive kinase inhibitor *p21* consistent with the activation of genotoxic stress pathways (Figure 4E), and increased expression of several oxidative stress response genes (Figure 4E).

In addition to the brain and spinal cord, we also investigated the retina, as retinal degeneration is a frequent symptom of CS and XP/CS patients [38], that is also reproduced in CSA- and CSB-deficient mice [23]. TUNEL staining revealed cell death in both the inner and outer nuclear layers of the retina of 4- and 14-week old *Xpg^−/−^* mice (Figure 4F). Hence, *Xpg^−/−^* mice display loss of photoreceptor cells as well as degeneration of the retinal circuitry. Together these data indicate the occurrence of widespread progressive degenerative changes in *Xpg^−/−^* nervous system, strongly resembling the phenotype of ERCC1-deficient mice.

### Liver-specific inactivation of XPG results in progeroid features in the liver without causing early death or reduced body weight

Transgenic expression of ERCC1 in the liver has been shown to alleviate growth deficiency and to extend lifespan of ERCC1-deficient mice [83], suggesting that liver abnormalities are an important determinant of the reduced lifespan of these mice. To determine the importance of liver pathology in the runting and the reduced lifespan of our *Xpg^−/−^* mice, we generated mice with liver-specific inactivation of the *Xpg* gene by crossing *Xpg^f/+^* mice carrying the floxed *Xpg* allele with heterozygous *Xpg^+/−^* mice that also express the *albumin-Cre* recombinase transgene that drives Cre expression specifically in hepatocytes [95] to yield *Xpg^f/−^*/*Alb-Cre^+^* mice, hereafter designated *Alb-Xpg* mice. This *Alb-Xpg* mouse also has the advantage that it allows to study the effect of liver XPG-deficiency in the absence of abnormalities in other tissues.

**Figure 5:**
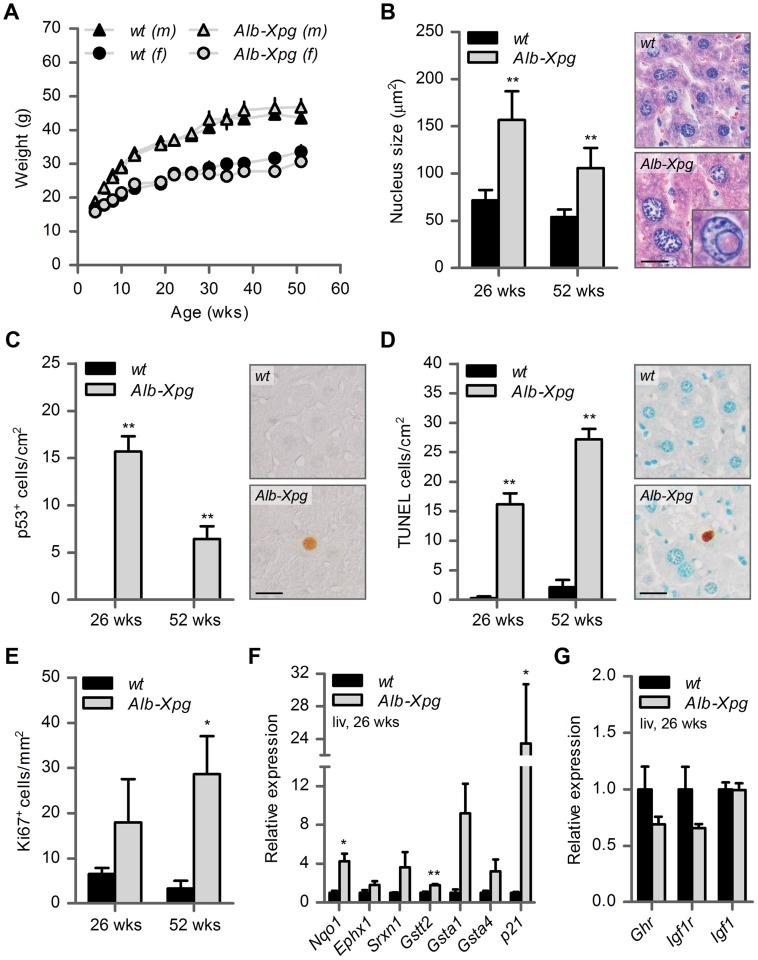
Aging features observed in the liver of liver-specific *Xpg* knockout mice. (A) Average body weight of C57Bl6/FVB F1 hybrid wild type (*wt*) males (black triangles), *wt* females (black circles), liver specific XPG-deficient (*Alb-Xpg*) males (gray triangles) and *Alb-Xpg* females (grey circles); n = 4 males/group, n = 2 females/group. (B) Average nucleus size of hepatocytes in the liver of 26- and 52-week old *Alb-Xpg* and *wt* mice; n = 4 animals/group. Bottom right: magnification of a nuclear inclusion found regularly in liver sections of 26- and 52-week old *Alb-Xpg* mice. (C) Quantification of p53-positive cells per cm^2^ in the liver of 26- and 52-week old *Alb-Xpg* and *wt* mice; n = 3 animals/group. (D) Quantification of TUNEL-positive cells per cm^2^ in the liver of 26- and 52-week old *Alb-Xpg* and *wt* mice; n = 3 animals/group. (E) Quantification of Ki67-positive cells per mm^2^ in the liver of 26- and 52-week old *Alb-Xpg* and *wt* mice; n = 3 animals/group. (F) Relative mRNA expression levels of several antioxidant genes and the DNA damage response gene *p21* in liver tissue of 26-week old *Alb-Xpg* and *wt* mice. All values are corrected for *TubG2, Hprt, and Rps9* expression and normalized to *wt* expression levels; n = 3 animals/group. (G) Relative expression levels of the somatotrophic genes *Ghr, Igf1r and Igf1* in liver tissue of 26-week old *Alb-Xpg* and *wt* mice. All values are corrected for *TubG2, Hprt, and Rps9* expression as internal standard and normalized to *wt* expression levels; n = 3 animals/group. Scale bars: 25 µm (B, C, D). Error bars indicate standard error of the mean. *p<0.05, **p<0.01.

A cohort of *Alb-Xpg* mice was allowed to reach the age of one year. All *Alb-Xpg* mice displayed normal growth and weight gain, and none of them died prematurely (Figure 5A). Livers of *Alb-Xpg* mice had an increased size compared to wild type, while brain, kidney and spleen displayed unaltered size and weight (Figure S5A). Albumin and glucose blood levels were the same as in control mice (Figure S5B and C). Histological analysis revealed anisokaryosis with karyomegaly in the liver of *Alb-Xpg* mice analyzed at 26 and 52 weeks (Figure 5B). The observed karyomegaly was more prominent than that observed in 14-week old *Xpg^−/−^* mice, and cells with intranuclear inclusions were more frequent. In addition, we identified p53-positive cells, increased cell death and increased cell proliferation in *Alb-Xpg* liver consistent with a progeroid degenerative phenotype (Figure 5C–E). Furthermore, real-time PCR showed that livers of *Alb-Xpg* mice displayed a massive induction of the DNA damage response gene *p21* as well as increased expression of several antioxidant effector genes (Figure 5F). We also observed a trend of reduced expression of *Ghr* and *Igf1r* (Figure 5G), hence, reproducing gene expression changes determined in livers of *Xpg^−/−^* mice. Activation of the *Nrf2* antioxidant response genes, reduction of the IGF1 axis, and increased proliferation shown by Ki67-staining are all consistent with liver regeneration after tissue damage [96]. As an additional control we showed that the expression of these genes is unaltered in livers from *Emx1-Xpg* mice that are XPG-deficient in the dorsal forebrain (see below; Figure S5D and E).

Together the data from the *Alb-Xpg* mice show that progeroid and gene expression changes in the liver triggered by the absence of XPG are not sufficient to explain the runted short-living phenotype of *Xpg^−/−^* mice.

### Dorsal forebrain specific inactivation of XPG results in a mixed macroglia/neuronopathy in cortex and hippocampus

**Figure 6:**
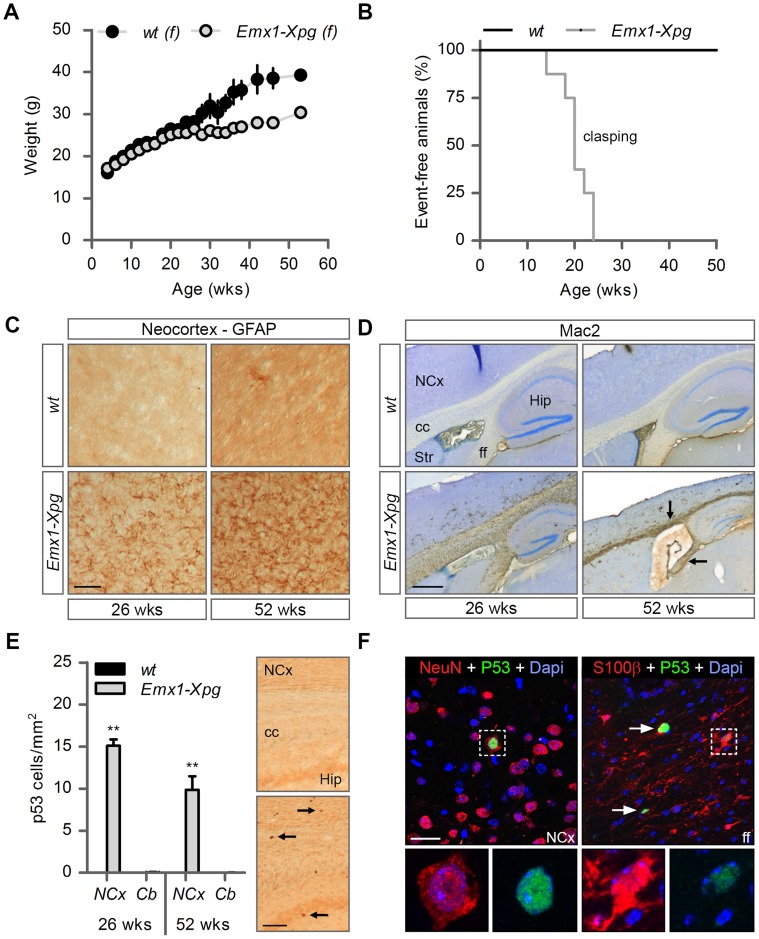
Age-related increase of neuronal stress in forebrain-specific *Xpg* knockout mice. (A) Average body weight of C57Bl6/FVB F1 hybrid wild type (*wt*) females (black circles) and forebrain-specific XPG-deficient (*Emx1-Xpg*) females (gray circles); n≥4 animals/group. (B) Onset of clasping of the hind limbs in *Emx1-Xpg* mice; n = 7 animals/group. (C) Representative images of GFAP immunostained sagittal neocortex sections of 26- and 52-week old *Emx1-Xpg* and *wt* mice showing progressive astrocytosis in *Emx1-Xpg* mice. (D) Representative images of Mac2 immunostained sagittal brain sections of 26- and 52-week old *Emx1-Xpg* and *wt* mice showing Mac2-positive microgliosis and a progressive decrease in size of the cerebral cortex and hippocampus of *Emx1-Xpg* mice. Arrows indicate microgliosis in corpus callosum and fimbria fornix. A thionin counterstaining was used. (E) Quantification of p53-positive cells in neocortex and cerebellum of 26- and 52-week old *Emx1-Xpg* and *wt* mice. Values are the average of four sections per genotype. Arrows indicate p53 positive cells. (F) Representative confocal images showing double labeled p53-NeuN cells in the neocortex (left) and p53-S100ß in the fimbria fornix (right) of 26-week old *Emx1-Xpg* mice. Arrows indicate p53 positive cells. NCx: neocortex, cc: corpus callosum, Str: striatum, ff: fimbria fornix, Hip: hippocampus. Scale bars: 50 µm (C), 500 µm (D), 200 µm (E) and 20 µm (F). Error bars indicate standard error of the mean. **p<0.01.

Our neuropathological analyses of *Xpg^−/−^* mice uncovered severe neurodegenerative changes at 14 weeks of age, compatible with neurological and motor deficits in these mice. Importantly, the presence of p53 in glia and abundant astrocytosis and microgliosis in the white matter indicate that abnormalities in *Xpg^−/−^* mice are not limited to neurons, but also involve glia cells. This is consistent with our findings in CS mouse models [62], [91], and with the neuropathological changes found in CS and XP/CS patients that is dominated by white matter pathology, in addition to neuronal, glial and vascular pathology, and, in severe cases, developmental abnormalities [39], [94], [97]–[99]. In previous studies, using a Cre-lox approach with *CamKIIα-Cre* and *L7-Cre* transgenic mice that drive Cre expression in post-mitotic forebrain neurons and Purkinje cells, respectively, we showed that neuron-specific deficiency of ERCC1 or combined deficiency of XPA and CSB was sufficient to trigger stochastic degeneration of these neuronal populations [89], [91], [92]. These studies showed that neurodegenerative changes in ubiquitous ERCC1- and XPA/CSB-deficient mice are not a consequence of developmental abnormalities, vascular problems, or degenerative changes in other organs. Furthermore, these neuron-specific mice enabled us to follow the degenerative process beyond the normal lifespan of the short-living ubiquitous ERCC1- and XPA/CSB-deficient mice [89], [91], [92]. In the present study we therefore used a similar approach, but with an *Emx1-Cre* transgenic line that drives Cre expression in progenitor cells of the dorsal telencephalon, to achieve inactivation of the *Xpg* gene not only in excitatory neurons of the neocortex and hippocampus, but also of astrocytes and oligodendrocytes in these brain areas [100]. Analysis of a cohort of *Emx1*-*Xpg* mice that were allowed to age for one year revealed no early death and showed that body weights were indistinguishable from that of wild type littermates until the age of 30 weeks. At older age the mean weight gain of *Emx1*-*Xpg* mice was significantly smaller than in control littermates (Figure 6A). This difference in weight was associated with a proportional reduced weight of internal organs (Figure S6A). Basal blood glucose concentrations were the same as in controls, indicating that reduced weight of old *Emx1*-*Xpg* mice is not a consequence of reduced energetic intake (Figure S6B).

To obtain a crude impression of the development of neurological symptoms, we examined the time of onset of clasping of the hind limbs when animals were lifted by their tails. This abnormality developed in *Emx1*-*Xpg* between 14–24 weeks of age and was not observed in control littermates (Figure 6B). The *Emx1*-*Xpg* mice did not develop tremors and deficits in accelerating rotarod performance (Figure S6C). Both the absence of these motor deficits and the delayed onset of clasping in comparison to the *Xpg^−/−^* mice can be explained by the selective inactivation of *Xpg* in neocortex and hippocampus, avoiding the bulk of circuitries controlling motor behavior in mice [101].

Macroscopic inspection of brains of *Emx1*-*Xpg* mice at 26- and 52-week already revealed that the neocortex was considerably smaller. Histological analysis confirmed that the neocortex was thinner, and showed that also the hippocampus was dramatically smaller, while other brain regions were unaltered (Figure S6D). GFAP immunohistochemistry showed a very strong increase in GFAP staining indicative of astrocytosis in both cortex and hippocampus (Figure 6C), and no changes in other brain regions. As in the *Xpg^−/−^* nervous system, astrocytosis was paralleled by microgliosis, identified by immunohistology with an Iba-1 antibody. Staining for Mac2 (also known as galectin-3), to outline phagocytosing microglia cells [102], revealed very high levels of Mac2-positive cells in the corpus callosum and the fimbria fornix of *Emx1*-*Xpg* mice (Figure 6D and S6D,E), and a moderate amount of phagocytosing microglia in the neocortex and hippocampus (Figure 6D). Remarkably, the presence of Mac2 could not be explained by axonal degeneration of cortical and hippocampal neurons solely, as we did not observe this phenomenon in our *CamKIIα-Ercc1* mice [89]. Furthermore, the capsula interna of *Emx1*-*Xpg* mice, which contains the descending corticofugal axons and shows severe axonal degeneration, did not show this dramatic increase of Mac2 staining (Figure 6D and S6D). Hence, the presence of high levels of Mac2 labelling may reflect the same phenomenon that we observed in our CS mice, i.e. the presence of phagocytosing microglia in the absence of axonal degeneration [91]. Accordingly, we also found an upregulation of Hsp25 expression in astrocytes in the corpus callosum and fimbria fornix (Figure S6F), a phenomenon that we also observed in the white matter of CS mouse models [91]. Furthermore, analysis of p53 expression in *Emx1*-*Xpg* showed that multiple p53-positive cells populated the fimbria-fornix and the corpus callosum (Figure 6E). Double-labelling of p53 with NeuN or the glia marker GFAP and S100β showed that some p53-positve cells were neurons (NeuN), but a large proportion were astrocytes (GFAP+, S100β+), or oligodendrocytes as identified on the basis of nuclear morphology (Figure 6F). Together the data indicate that *Emx1*-*Xpg* mice develop a combined neuro- and gliopathy of cortex and hippocampus.

## Discussion

XPG is a 3′-endonuclease that is essential for the excision step of the GG-NER and TC-NER subpathways of NER and that has additional, yet incompletely defined, roles in other repair processes and possibly in transcription [15], [16], [31], [53], [103]–[105]. Consistent with multiple diverse functions, mutations in XPG cause a spectrum of disease phenotypes varying from the UV-sensitivity disorder XP to the multisystem developmental/degenerative disorders XP/CS and COFS [52]–[55], [57]. Several *Xpg* mouse models have been generated that partially recapitulate the spectrum of genetic defects and disorders found in patients [63]–[66]. To better define the effect of the complete absence of XPG, in the present study we have generated mice with a Cre-inducible *Xpg* knockout allele and examined the phenotypes of total (designated *Xpg^−/−^*), liver-specific (*Alb-Xpg*), and dorsal forebrain-specific (*Emx1-Xpg*) XPG-deficient mice. Consistent with previous data [63] we found that *Xpg^−/−^* mice are born with normal size and weight, but progressively fail to grow and to gain weight after birth, combined with a short lifespan. In addition, we found that lifespan was influenced by genetic background, in that *Xpg^−/−^* mice displayed a much shorter lifespan in C57BL6 background (3 weeks), compared to FVB or C57BL6/FVB F1 hybrid background (16–18 weeks). An effect of genetic background is well known for many mouse models of disease and was also noted in our *Ercc1*-mutant mice [51], [72]. Differences in genetic background likely explain the differences in lifespan of our C57BL6/FVB F1 hybrid *Xpg^−/−^* mice and the *Xpg^−/−^* mice of Harada and coworkers [63], which all died at 3–4 weeks of age similar to our C57BL6 *Xpg^−/−^* mice. However, what mechanism defines the short lifespan of our C57BL6 *Xpg^−/−^* mice remains to be determined. Importantly, the effect of genetic background in mice suggests that genetic context may also impact the disease phenotype in CS and XP/CS patients. Interestingly, preliminary analysis of neuropathological changes of our C57BL6 *Xpg^−/−^* mice at 3 weeks of age revealed subtle degenerative changes (Figure S7) that are comparable in severity to the neurodegenerative changes of C57BL6/FVB *Xpg^−/−^* mice of 4 weeks of age, and considerably milder than those in 14-week old C57BL6/FVB F1 hybrid *Xpg^−/−^* mice (compare Figure S7 with Figure 4). These data suggest time-dependence of the neurodegenerative phenotype and indicate that the much shorter lifespan of C57BL6 *Xpg^−/−^* mice cannot be explained by enhanced neurodegeneration. An alternative possibility suggested by the data of Harada and coworkers [63] is that C57BL6 *Xpg^−/−^* mice display an exaggerated developmental phenotype with more prominent developmental deficits, such as an improperly developed gastro-intestinal tract, that would reduce their ability to cope with extra-uterine life. Recent evidence from XPA/CSA-deficient mice bred in C57BL6 background suggest that providing mutant pups with soft food before weaning is sufficient to increase survival from 3–4 weeks to 12–18 weeks [106]. So far, we found no beneficial effect of soft food in our C57BL6 *Xpg^−/−^* mice, but this remains to be further examined. Nevertheless, together the data suggest that the ability of short-living NER mutant mice to survive beyond weaning might also depend on the ability to cope with the transition from mother milk to other food.

### *Xpg^−/−^* mice as a progeria model

The main goal of the present study was to determine the extent to which XPG deficiency in mice results in multisystem progeroid degenerative changes as observed in CS and XP/CS patients, as well as in other NER-deficient mouse models including *Xpa/Csb*, *Xpd*, and *Ercc1* mutants [48], [51], [68], [69], [72], [77], [107]. Our data show that, although seemingly normal at late embryonic stage, and showing only mild degenerative features at 4 weeks of age, C57BL6/FVB *Xpg^−/−^* at 14–16 weeks showed abundant degenerative changes in multiple tissues, indicative of accelerated aging. These degenerative/accelerated aging features included kyphosis, cachexia, osteoporosis, liver aging, and abundant nervous system degenerative changes. In addition, the data from our liver- and dorsal forebrain-specific *Xpg* mice showed that a much more severe tissue-specific degenerative phenotype could be obtained in these mice because of survival beyond the maximal lifespan of *Xpg^−/−^* mice. Hence, the data indicate that *Xpg^−/−^* mice indeed develop a multisystem premature aging phenotype reminiscent of the phenotypes of some of our other NER-deficient mouse models, in particular the *Ercc1^Δ/−^* mice, which show a slightly longer lifespan and comparable pathological changes in liver and nervous system (see below). Moreover, the *Xpg^−/−^* mouse phenotype shares many features with XP-G/CS patients [17], [94], [108] including a cachectic ‘frail’ appearance with loss of subcutaneous fat and signs of osteoporosis (Table 2).

**Table 2:** Comparison of the *Xpg^−/−^* phenotype to that of XP/CS patients.

Phenotypic characteristics	XPG mouse	XP(G)/CS patient
***UV sensitivity***		
Cellular level	y	y
Organismal level	nd	y
Sensitivity to oxidative damage-cell	n	y
***Growth and lifespan***		
Growth retardation	y	y
Reduced subcutaneous fat	y	y
Reduced lifespan	y	y
***Sensory systems***		
Retinal degeneration	y	y
Sensorineuronal hearing loss	nd	y
***Bones***		
Osteoporosis	y	y
Kyphosis	y	y
***Nervous system***		
Gait ataxia	y	y
Action tremor	y	y
Cognitive decline	y	y
Brain calcifications	nd	y
Ventricle enlargement	y	y
White matter abnormalities	nd	y
Cerebral and cerebellar atrophy	y	y
Astrogliosis	y	y

y = present, n = not present, nd = not determined.

### Compensatory survival response in *Xpg^−/−^* liver

Previously we have shown that transcription-blocking lesions trigger a “survival response” involving somatotroph attenuation and increased resistance to oxidative stress, which resembles the response triggered by dietary restriction and is associated with delaying many aspects of aging and increasing lifespan [51], [68], [84]. This mechanism has been observed in response to direct but persistent DNA damage as well as during the course of aging, both in natural aging as well as in specific progeroid mouse models, and is activated in long-lived mutant mice and centenarians [51], [68], [109]. A central hallmark of this survival response is the downregulation of genes involved in the GH/IGF axis, combined with upregulation of genes involved in the antioxidant response. Using real-time PCR for selected genes of these pathways in the present study, we show that *Xpg^−/−^* mice display survival-response-like changes in gene expression in liver cells, including a decrease in *Ghr* mRNA levels and a robust increase in expression of the antioxidant defense effector genes *Nqo1, Srxn1, Gstt2, and Gsta1*. Similar changes were observed in the liver of liver-specific XPG-deficient mice. Consistent with previous observations concerning the survival response, we found significantly reduced circulating glucose levels in the *Xpg^−/−^* mouse. However, we found no change in glucose in the liver-specific mouse, indicating that reduced glucose levels are not necessarily a consequence of gene expression changes in the liver. We did not observe significantly lower levels of *Igf1* and *Igf1r*, as previously found for several other progeroid DNA repair mutants [51], [84], although we did find a trend of reduced expression.

Increased expression of antioxidant genes was also observed in the central nervous system (i.e. cerebellum) of *Xpg^−/−^* mice, but the identity and the degree of changed expression is somewhat different. For instance, *Nrf2* shows a relatively increased expression in cerebellum but not in liver, while *Nqo1* shows a very large relative increase in liver, and a modest relative increase in cerebellum. These differences may be explained by altered stress responses in different cell types [110]. In addition, in the nervous system, changes in gene expression may result from the death and disappearance of neurons and reactive proliferation of glial cells and as a consequence, reduced and increased expression of neuronal and glial genes, respectively.

### Progeroid features in the livers of *Xpg^−/−^* animals are not sufficient to cause reduced growth and lifespan

The liver of *Xpg^−/−^* mice showed several characteristics also found in aging liver, i.e. increased nuclear size and the presence of nuclear inclusions. These changes were much more prominent and associated with additional degenerative changes in liver-specific XPG-deficient mice. However, despite prominent liver pathology, the liver-specific *Xpg^−/−^* mice at 52 weeks did not show altered weight, nor altered glucose and albumin levels suggestive of metabolic problems. Although at this point we cannot exclude that our liver-specific mutants will develop health problems in their second year of life and might develop a shorter than normal lifespan, it is safe to conclude that liver problems on their own cannot be the main culprit of the reduced lifespan and the small cachectic appearance of *Xpg^−/−^* mice. In contrast to our data, findings in *Ercc1* mutant mice show that transgenic overexpression of ERCC1 in the liver alleviates growth deficiency and extends lifespan [83], indicating that liver abnormalities are an important determinant of the reduced lifespan of these mice. A possible explanation is that liver pathology is more prominent in *Ercc1* mutant mice, putatively as a consequence of additional defects in interstrand crosslink and double-strand break repair [48]. An alternative explanation for the differences in results could be that the impact of liver pathology on survival depends on the severity of pathology in other organs. This hypothesis is testable by studying survival of liver-specific ERCC1-deficient mice, and *vice versa* by generating liver-corrected *Xpg^−/−^* mice using a transgenic ‘rescue’ strategy as reported by Selfridge et al. [83]. A possible conclusion from these studies could be that *Ercc1* and *Xpg* mutant mice die prematurely as a consequence of a synergistic deleterious effect of multi-organ failure. In view of the severe neurological and neurodegenerative deficits in 14–16 week old *Xpg^−/−^* mice, yet an alternative scenario is that *Xpg^−/−^* mice die as a consequence of nervous system abnormalities (see below). This scenario is realistic for a subset of human NER-deficient patients, in particular those developing XP with neurological abnormalities [36], [40]. Interestingly, our dorsal forebrain-specific XPG-deficient mice also showed reduced weight-gain after 30 weeks of age. At this age these mice displayed severe neuronal degeneration in neocortex and hippocampus, as a result of which the mice would be expected to have severe cognitive deficits, whereas basal motor functions remain unaltered. Weight loss was not associated with altered glucose levels or gene expression changes in the liver (Figures S5 and S6). We noted the same reduced weight-gain in our forebrain neuron-specific XPA/CSB-deficient mouse model [91]. The data suggest that severe disruption of cortical or hippocampal circuitries may result in the weight loss, but the precise mechanism remains to be defined.

### XPG deficiency causes age-dependent degeneration of neurons and macroglia

Our data show that *Xpg^−/−^* mice, within their relatively short lifespan (16–18 weeks), develop neurological abnormalities of increasing severity in association with degenerative changes throughout the nervous system. These nervous system degenerative changes seemingly are more severe than those previously reported for another line of *Xpg^−/−^* mice [111]. The differences in nervous system neurodegenerative changes can be explained by the very short lifespan (3 weeks) of previously reported *Xpg^−/−^* mice, since our C57BL6 *Xpg^−/−^* mice, which show the same short lifespan, also displayed a low level of nervous system pathology at the end of life (see above and Figure S7). Together with our demonstration that dorsal forebrain-specific XPG-deficient mice, allowed to age for one year, display very severe neurodegenerative changes, our data indicate that age is a key determinant in the development of neurodegenerative changes in *Xpg^−/−^* mice. Our data also indicate that XPG deficiency does not result in obvious neurodevelopmental abnormalities, although subtle developmental abnormalities cannot be excluded at this point.

The neurodegenerative changes in our *Xpg^−/−^* mice strongly resemble those of incomplete ERCC1-deficient *Ercc1^Δ/−^* mice that have a slightly longer lifespan (24–30 weeks). Our studies with *Ercc1^Δ/−^* mice and neuron-specific *Ercc1* knock-out animals indicate that ERCC1-deficient neurons in time stochastically accumulate structural and functional deficits to eventually die and disappear [89], [90], [92], [112]. A stochastic accumulation of deficits also appears to occur in *Xpg^−/−^* neurons. Thus, the widespread distribution of astrocytosis and microgliosis, as well as p53 and TUNEL staining, indicates that degenerative changes in the *Xpg^−/−^* nervous system affect all neuronal populations. The increased size and frequency of axonal spheroids in Purkinje cells, and a diversity of Golgi apparatus morphological abnormalities in motor neurons, on the other hand, illustrate that *Xpg^−/−^* neurons may asynchronously accumulate a variety of degenerative features over time. This widespread and asynchronous accumulation of cellular damage in *Xpg^−/−^* neurons is consistent with a model in which neurons are afflicted by stochastic DNA lesions that deregulate gene expression, as we have proposed for *Ercc1^Δ/−^* mice [89]. A prime role for genotoxic stress in causing the degenerative phenotype in the *Xpg^−/−^* nervous system is further suggested by the presence of stochastically distributed p53-positive cells and increased expression of the DNA damage responsive *p21* gene.

Together the degenerative changes in the *Xpg^−/−^* nervous system favor a pathogenic model involving deficient DNA repair in the same way as proposed for ERCC1-deficient mice [48], [89]. The central role of DNA damage in the *Xpg^−/−^* nervous system raises questions about the identity of the DNA lesions involved. Importantly, the absence of a significant neurodegenerative phenotype in entirely NER-deficient *Xpa^−/−^* mice has led to the conclusion that DNA lesions that accumulate in the nervous system in the absence of NER are not sufficient to trigger neuronal degeneration within the normal lifespan of mice [74], [91], [113]. In the case of ERCC1-deficient mice, it therefore has been proposed that the degenerative nervous system changes result from combined deficiencies in NER and other DNA repair pathways, i.e. interstrand crosslink repair and double-strand break repair [48], [89]. It is possible that a similar mechanism may contribute to the severe neurodegenerative phenotype caused by XPG deficiency, as XPG has been reported to play a role in repair of crosslinks induced by mitomycin C [114]. An alternative possibility is that XPG deficiency reproduces the severe degenerative phenotype resulting from crossing NER-deficient *Xpa^−/−^* with the *Csb^−/−^* or *Csa^−/−^* CS mouse models [67]–[69], [91], [106]. In this scenario XPG deficiency combines the deleterious synergistic interaction between NER deficiency and loss of the yet incompletely understood non-NER activities that underlie CS. CS proteins operate together at the interface of DNA repair and transcription regulation, and several mechanisms have been put forward to explain CS symptoms [46].

XPG can interact directly with both CSB and RNA Polymerase II [31], and may be implicated in the repair of transcription blocking oxidative lesions, for instance by recruiting base excision repair factors [32]–[34]. Accordingly, cells from CS patients including XP-G/CS cells have been found to display increased vulnerability to inducers of oxidative DNA lesions [55], [115], [116]. Importantly, Soltys et al. showed oxidative damage sensitivity of XP-G/CS, but not of XP-G patient cells [55]. Yet, in accordance with findings by Harada and coworkers [63], we found that cells from our *Xpg^−/−^* mice do not display increased vulnerability to inducers of oxidative DNA lesions such as KBrO_3_ (Figure S1B). This is despite clear indications of endogenous damage in, for example, the retina of these mice, as has been previously observed for *Csb/Xpa* mice that are sensitive to oxidative damage [23]. It is currently unclear whether this is a peculiarity of these particular mouse cells in culture, as we have reported that cells from CSB-deficient mice are sensitive to IR and paraquat [117], [118]. It has been argued that cultured cells may build up defense responses that mask the increased vulnerability of these cells *in vivo* [119].

To what extent do *Xpg^−/−^* mice reproduce the nervous system abnormalities of patients carrying XPG mutations? Roughly, the progressive widespread neurodegenerative changes of *Xpg^−/−^* mice are reminiscent of neuropathological changes of patients with ‘XP-type neurological degeneration’ [39], [40], [97], [120]. In well documented cases these patients, carrying XPA mutations resulting in complete NER deficiency, develop a wide array of neurological symptoms that show early juvenile onset, over time become more severe, and ultimately cause premature death in mid-adult life [39], [40], [97]. However, the limited documented cases indicate that XP-G patients either develop no neurological symptoms, or reproduce mild to severe neurological and neuropathological features of CS [39], [40], [57], [94], [120]. In CS and XP/CS patients, neuronal degeneration generally is less prominent. Instead, these patients, including documented XP-G/CS cases, show prominent white matter degeneration, vascular pathology, calcium depositions, and, in severe cases, developmental abnormalities [39], [94], [97]–[99]. We have recently noted that CSA- and CSB-deficient CS mouse models, in addition to mild neurodegenerative changes, develop subtle white matter abnormalities and glial pathology reminiscent of the glia and white matter degenerative changes of CS patients, albeit milder [62], [91]. The higher levels of neuronal degeneration in *Xpg^−/−^* mice hamper detection of primary glial and white matter pathology, due to secondary glial pathology caused by neuronal degeneration. However, the severe white matter pathology in the corpus callosum and fimbria-fornix in our dorsal forebrain specific XPG-deficient mice strongly indicates that XPG deficiency triggers CS-like white matter pathology in mice.

### Concluding remarks

In this study, we show that *Xpg^−/−^* mice from young age onwards develop a multisystem degenerative phenotype and die before the age of 20 weeks. This phenotype strongly resembles the progeroid features of CS and XP/CS patients. In addition, the *Xpg^−/−^* mouse model shows a number of similarities to other NER-based mouse models of progeria such as *Xpa/Csb* and *Ercc1* mutants [3], [62], pointing to the importance of NER in multiple tissues. In particular, a detailed analysis of commonalities and differences between *Xpg^−/−^* and *Ercc1* mutant mice may aid in our understanding of the contribution of different types of DNA damage and DNA repair defects in the accelerated aging process, since both endonucleases have a joint role in the damage excision step of NER but have divergent additional non-NER roles. Together our findings further stress the relationship between compromised DNA repair and acceleration of specific aging features, as well as progressive neurodegeneration. Finally, the neurodegenerative phenotype indicates that *Xpg^−/−^* mice may serve as a model to test intervention strategies aimed at reducing the formation of detrimental DNA lesions in neurons.

## Materials and Methods

### Generation of a floxed *Xpg* allele

The *Xpg* targeting construct was generated using multiple elements. First, a cassette consisting of a Neomycin (NEO) resistance marker, flanked by *Frt* sites, and followed by a single *LoxP* site was cloned into a modified pBlueScript SK+ vector containing a PGK-DTA negative selection marker, making use of a klenow blunted ApaI (insert)/XbaI(vector) and a NotI restriction site. Second, *Xpg* homologous arms were PCR amplified from C57BL6 genomic DNA (originating from BAC clone RP24-343K18) and cloned into the same plasmid. The following primers (non-homologous regions indicated in italics; the *LoxP* sequence is underlined) were used for amplification of the 5′ and 3′ arm, respectively: LAF2 (5′-*CGCACCCGGG*TGTGATCCTGTGGTCCTGTAGT-3′) and LAR2 (5′-*CCATCGAT*ATCCTCAGAAAGGTATCTCTTAAGCA-3′), yielding a 3.2-kb XmaI-ClaI fragment; RAF1 (5′-*CCCTGCTAGC*GGGATGAGGAATCGTGACTAAGGAG-3′) and RAR1 (5′-*CCGCAGCGGCCGC*AAACAAGGGACCCAAATGTAGGCT-3′), yielding a 2.0-kb NheI-NotI fragment, where the restriction sites were introduced in the PCR primers. Last, the third exon of *Xpg* followed by a PCR-introduced *LoxP* site was amplified using the primers Ex3LoxF2 (5′-*GGGAACCG*GTTTGAGTGTCCTTGGTGACAGG-3′) and Ex3LoxR2 (5′-*CCCTGCTAGC* ATAACTTCGTATAGCATACATTATACGAAGTT ATCC-3′), yielding a 350-bp AgeI-NheI fragment, which was inserted between the neomycin cassette and the 5′ homology arm.

Next, a total of 10 µg of NotI-linearized targeting vector was electroporated to Ola129 ES cells, and the targeted clones were selected through the use of the Neomycin selection marker (G418 200 µg/ml). Clones resistant for G418 were initially screened by PCR, using a forward primer in exon 3 (F3 5′-GAGACAGGCTCTGAAAACTGCTT-3′) and a reverse primer outside the 3′ homologous region (R3 5′-CACTGAACAAACAAGGGACCCAAA-3′). ES clones showing a 2.2-kb fragment in addition to the wild type 2.3-kb fragment after NheI digestion of the PCR product were further screened by Southern blot. ES genomic DNA was digested with EcoRI and hybridized with a 0.9-kb DpnI restriction fragment from BAC RP24-343K18, spanning the 2^nd^ exon of *Xpg*. The probe hybridizes to a 7.4-kb fragment in wild type DNA and to an additional 4.1-kb fragment in targeted DNA.

ES cells from two independent targeted clones were micro-injected into C57BL6 blastocysts. Heterozygous mutant mice were generated by crossing the male chimeras with C57BL6 females and verified by coat color and PCR genotyping. The Neomycin (NEO) resistance gene was flanked by *Frt* sites to allow specific elimination of this dominant selectable marker by an *Flp* recombinase to avoid potential undesired influence of the *Neo* gene on *Xpg* transcription or mRNA processing. The NEO cassette was removed by crossing mice carrying the targeted allele with *Cag-Flp* recombinase FVB/N transgenic animals [121]. These mice carry the floxed allele, and are referred to as *Xpg^f^* throughout this paper. Thereafter, the F_3_ offspring was crossed with a *Cag-Cre* C57BL6 transgenic [73], resulting in Cre-mediated recombination and excision of the third exon. *Xpg^+/−^* animals were backcrossed to C57BL6 and FVB/N in parallel, at least ten times, and interbred to obtain C57BL6, FVB/N and C57BL6/FVB F1 hybrid *Xpg^−/−^* mice. To achieve liver specific *Xpg* gene inactivation, a transgenic line with Cre recombinase under the control of the albumin promoter (hereafter referred to as *Alb-Cre*) was used [95]. Female *Alb-Cre^+^* mice were crossed with male *Xpg* ^+/−^ mice (both in a C57BL6 background). Female *Xpg^+/−^ Alb-Cre^+^* mice, obtained from these breedings, were crossed with male *Xpg* ^f/f^ FVB/N mice to yield hybrid *Xpg* ^f/−^ *Alb-Cre^+^* mice. *Xpg* ^f/−^ *Alb-Cre^+^* mice (in a C57BL6/FVB F1 hybrid background) are heterozygous for *Xpg* in their entire body, except for the hepatocytes in the liver, which are homozygous for *Xpg* after Cre excision of the floxed allele. All littermates, with and without Cre-recombinase expression were used as controls (referred to as *wt*).

FVB/N *Xpg^f/f^* animals were similarly bred to the female offspring from C57BL6 *Xpg* ^+/−^ and C57BL6 *Emx1-Cre* mice [100] to obtain forebrain specific XPG knock-out animals (referred to as *Emx1-Xpg*). All animals used in the studies described in this paper were of the same C57BL6/FVB F1 hybrid background (unless otherwise stated) and had *ad libitum* access to water and standard mouse food (CRM pellets, SDS BP Nutrition Ltd.; gross energy content 4.39 kcal/g dry mass, digestible energy 3.2 kcal/g or AIN93G synthetic pellets, Research Diet Services B.V.; gross energy content 4.9 kcal/g dry mass, digestible energy 3.97 kcal/g). Since the *Xpg* knockout animals were smaller, food was administered within the cages and water bottles with long nozzles were used from around two weeks of age. Experiments were performed in accordance with the Principles of Laboratory Animal Care (National Institutes of Health publication no. 86-23) and with the guidelines approved by the Erasmus University Animal Care Committee.

### Genotyping

For PCR genotyping the following primers were used: F1 forward primer (5′-TCTGTTTAGGTGGTGCCCATTT-3′) annealing 5′ of the third exon of *Xpg*; NeoF forward primer (5′-GCTTCCTCGTGCTTTACGGTAT-3′) located in the Neomycin resistance marker; R1 reverse primer (5′-CGACAGCACTTCTTTCTCCTTAGT-3′) annealing 3′ of the third exon of *Xpg*. A 711-bp fragment was generated from the targeted allele using primers NeoF and R1, whereas a 495-bp and 227-bp fragment are amplified from the wild type and knockout allele, respectively, using the F1/R1 primerset. Cycling conditions were 95°C for 45 sec, 58°C for 45 sec, 72°C for 1 min (35 cycles), followed by an extension at 72°C for 5 min.

### Sequencing

Total RNA was extracted from wild type and *Xpg^−/−^* liver using TRIzol reagent and reverse transcribed with SuperScript II Reverse Transcriptase (Life technologies), according to the manufacturer's instructions, to generate cDNA. A 0.4-kb PCR fragment ranging from the 2^nd^ to 6^th^ of *Xpg* was produced using the following primers: Ex2F (5′-GCTCATCTTCTCACATTATTCC-3′) and Ex6R (5′- GGTAAACTCTTTCATGTCAGTC-3′) and analyzed by Sanger sequencing.

### Phenotype scoring

The mice were weighed and visually inspected weekly, and were scored for the onset of various phenotypical parameters. Clasping was measured by suspending mice from their tails for 20 seconds. A clasping event was scored when retraction of both hind limbs towards the body was observed for at least 5 seconds. Whole body tremor was scored if mice were trembling for a combined total of at least 10 seconds, when put on a flat surface for 20 seconds. Mice showing an abnormal curvature of the spine were scored as having kyphosis.

### Isolation and analysis of MDFs

Primary MDFs were isolated from the tail of 12–14 week old *Xpg* ^−/−^ animals and wild type littermates. Minced tail skin was immersed in F10/DMEM culture medium supplemented with 20% fetal calf serum, 50 µg/ml penicillin/streptomycin, and 1.6 mg/ml type II collagenase (Gibco, Life Technologies). After incubation at 37°C for 24 hours, MDFs were filtered through a 40 µm cell strainer, centrifuged for removal of the collagenase, and cultured at 37°C, 5% CO_2_, and 3% O_2_.

### Immunoblot analysis of XPG protein in MDFs

Whole cell extracts were prepared from cultured MDFs using cells isolated from four wild type mice and four *Xpg^−/−^* littermates. Proteins from 30 µl of each extract were separated by electrophoresis on 7% SDS-PAGE gels and transferred overnight onto a nitrocellulose membrane. As previously described [31], XPG protein was detected with a rabbit polyclonal antibody designated R2 (97727) that was raised against a conserved peptide from the spacer region (R-domain) of XPG corresponding to residues 267–281 of the human protein, which are identical with the same residues in mouse XPG except for amino acid 267, which is E in the human protein but Q in mouse. Whole cell extract from human embryonic kidney 293 cells was used as a positive control for XPG protein. As a loading control, tubulin was detected using a commercial antibody.

### Cell survival assays

MDFs were seeded in triplicates at equal densities and treated 24 h after seeding as indicated. After 48 h recovery cell survival was determined by cell count on Beckman Coulter, Z2 Coulter particle count and size analyzer.

### Unscheduled DNA Synthesis (UDS)

UDS was performed using the Click-iT EdU imaging kit (Life technologies). MDFs were seeded on coverslips and 24 h later washed with PBS and irradiated with 16 J/m^2^ UV-C (Philips) or mock treated. Cells were directly labelled for 3 h in thymidine-free Ham's F10 medium supplemented with 10% dialyzed serum, 50 µg/ml penicillin/streptomycin, 20 µM Ethynyl-deoxyuridine and 1 µM Fluoro-desoxyuridine. After a PBS wash, the cells were chased with 10 µM non-labelled thymidine in normal medium for 15 minutes and fixed with 3.7% formaldehyde. Slides were washed 3× with PBS/3%BSA and permeabilized with PBS/0.5%Triton-X100 for 20 min. The Click-iT reaction, linking azide-conjugated Alexa dye to ethynyl groups was performed for 30 min in a dark, humid environment. After 3× PBS/3%BSA and 2× PBS wash, the slides were mounted with vectashield containing DAPI (Vector Laboratories) to stain nuclei.

### Recovery of RNA Synthesis (RRS)

RRS was performed using the Click-iT EU imaging kit (Life Technologies). MDFs were seeded on coverslips and 8 h later washed with PBS and irradiated with 16 J/m^2^ UV-C (Philips) or mock treated. After 14 h recovery, the cells were labelled for 2 h in 0.1 mM EU-containing medium (Ham's F10 medium supplemented with 10% dialyzed serum, 50 µg/ml penicillin/streptomycin and 20 mM HEPES). After a PBS wash, the cells were fixed and processes as described for UDS.

### Mechanical testing of bone strength

Force-deflection curves from the left femora of 14-week-old and 16-week-old mice were acquired in a three-point bending assay using a Chatillon TCD series mechanical test frame (Technex BV, The Netherlands), equipped with 3 mm hemi-cylindrical supports with a 8.5 mm total span. Width between the supports was adjusted according to the anatomical landmarks of the femur, i.e. lesser trochanter and condyles. The femora were aligned such that the femoral head was in the horizontal plane and the posterior aspect of the condyles was facing down. All samples were preconditioned for five cycles to 2 Newton (N) at a rate of 0.6 mm/min before testing to failure at a rate of 0.1 mm/min. The obtained force-deflection curves were analyzed for bone strength (N/mm), which was represented by the Δ force/deflection of the linear part of the curve.

### Micro-computed tomographic (micro-CT) quantification of bone thickness

*Xpg^−/−^* and wild type mice were sacrificed by cervical dislocation at scheduled ages (7, 14, 16, 18 weeks), femora were excised and non-osseous tissue was removed. Left femora were placed in PBS and stored at −20°C until further use for mechanical testing and the right femora were fixated (4% formalin). Two days post-fixation, the right femora were scanned using the Skyscan 1076 in vivo X-Ray computed tomography (Skyscan, Kontich, Belgium) with a voxel size of 8.88 µm. Osseous tissue was distinguished from non-osseous tissue by segmenting the reconstructed grayscale images with an automated algorithm using local thresholds [122]. The region of interest (ROI), i.e. distal metaphysis of the femora, was selected by using 3D data analysis software. To compensate for bone length differences between the *Xpg^−/−^* and wild type mice, the length of each ROI was determined relative to the largest specimen femur of the cohort.

Cortex and trabeculae of the metaphysis were separated using in-house developed automated software. Thickness of the trabeculae and cortices were assessed using 3D analysis software as described [123] using the CT analyzer software package (Skyscan). A bone specimen with known bone morphometrics was included within each scan as a quantitative control.

### Real-time PCR

TRIzol reagent (Life Technologies) was used to isolate total RNA from mouse tissue specimens. 4 µg RNA was reverse transcribed using SuperScript II (Life Technologies). Real-time PCR was performed on a Bio-Rad CFX96 thermocycler using SYBR Green (Sigma-Aldrich) and Platinum Taq polymerase (Life Technologies). Generation of specific PCR products was confirmed by melting-curve analysis and gel electrophoresis. For data analysis, the induction of target cDNA was calculated by the method described by [124]. p-values were determined using two-tailed t-tests. The used gene specific real-time PCR primers are listed in Supplementary Table S1.

### Blood glucose and albumin levels

Mice were euthanized by CO_2_ asphyxiation and blood was immediately collected from the heart. Glucose levels were measured using a Freestyle mini blood glucose meter. Albumin levels were measured in blood plasma using a mouse albumin ELISA kit (Immunology Consultants Laboratory, Inc.).

### Antibodies

Primary antibodies (supplier; dilutions) used in this study were as follows: rabbit anti-Calbindin (Swant; 1∶10,000); goat anti-ChAT (Millipore; 1∶500); rabbit anti-GFAP (DAKO; 1∶8,000); mouse anti-GM130 (BD Transduction; 1∶100); rabbit anti-Hsp25 (Enzo; 1∶8,000); rabbit anti-Iba-1 (Wako; 1∶5,000); rat anti-Ki67 (DAKO; 1∶200); rat anti-Mac2 (Cedarlane;1∶2,000); mouse anti-NeuN (Millipore; 1∶1,000); rabbit anti-p53 (Leica; 1∶1,000). For avidin– biotin–peroxidase immunocytochemistry biotinylated secondary antibodies from Vector Laboratories, diluted 1∶200 were used. FITC-, Cy3-, and Cy5-conjugated secondary antibodies raised in donkey (Jackson ImmunoResearch) diluted at 1∶200 were used for confocal immunofluorescence.

### Histological procedures

TUNEL staining on liver, brain and retina: To quantify apoptotic cells, specimens were fixed overnight in 10% buffered formalin, paraffin-embedded, sectioned at 5 µm, and mounted on Superfrost Plus slides. Paraffin sections were employed for TdT-mediated dUTP Nick-End Labeling (TUNEL) assay using a commercial kit (Apoptag Plus Peroxidase in situ apoptosis detection kit, Millipore). Sections were deparaffinized and incubated as described by the manufacturer. HE staining on liver, skin and small intestine: Liver, skin and intestine specimens were processed using the same fixation and sectioning methods as described for the TUNEL staining. Paraffin sections were deparaffinized, rehydrated in decreasing concentrations of ethanol and stained with haematoxilin and eosin.

For immunohistochemistry, paraffin sections of liver and intestine specimens were deparaffinized, rehydrated in decreasing concentrations of ethanol, treated for 10 minutes with 3% H_2_O_2_ to quench endogenous peroxidase activity and heated to 100°C for 1 h in 10 mM sodium citrate buffer, pH 6, for antigen retrieval. The amount of damaged (p53) and proliferating (Ki67) cells were subsequently detected using the avidin–biotin–immunoperoxidase complex method (ABC, Vector Laboratories, USA) with diaminobenzidine (0.05%) as chromogen.

Gelatin sections of brain and spinal cord: Mice were anaesthetized with pentobarbital and perfused transcardially with 4% paraformaldehyde. Brain and spinal cord were dissected out, post-fixed for 1 h in 4% paraformaldehyde, cryoprotected, embedded in 12% gelatin, rapidly frozen, and sectioned at 40 µm using a freezing microtome or stored at −80°C until use. Frozen sections were processed free floating using the ABC method (ABC, Vector Laboratories, USA) or single-, double-, and triple-labelling immunofluorescence. Immunoperoxidase-stained sections were analyzed and photographed using an Olympus BX40 microscope. Immunofluorescence sections were analyzed using a Zeiss LSM700 confocal.

### Rotarod performance

Average time spent on an accelerating rotarod (Ugo Basile). *Xpg^−/−^* mice and wild type controls were given four consecutive trials of maximally 5 minutes with inter-trial intervals of 1 hour. *Emx1-Xpg* mice were given two trials per day with a 1 hour inter-trial interval for four consecutive days.

### Grip strength

Grip strength was determined by placing mice with forelimbs or all limbs on a grid attached to a force gauge, and steadily pulling the mice by their tail. Grip strength is defined as the maximum strength produced by the mouse before releasing the grid. For each value the test is performed in triplicate.

### Statement of ethical approvement

This study was performed in strict accordance with the recommendations in the Guide for the Care and Use of Laboratory Animals of the National Institutes of Health. The protocol was approved by the Committee on the Ethics of Animal Experiments of the Erasmus MC (Permit Numbers: 139-03-08, 139-09-03, 139-12-18).

## Supporting Information

Figure S1 (A) Recombination of *Frt* and *LoxP* sites yield a *Xpg^−/−^* genetic status with a premature STOP codon in exon 3 as shown by Sänger sequencing of mRNA isolated from *Xpg^−/−^* liver tissue. (B) Primary *Xpg^−/−^* and wild type (*wt*) MDFs, cultured at low (3%) O_2_ levels were treated with the indicated doses of KBrO_3_ (left) or cisplatin (right) for 1 h. After 48 h recovery, survival was assessed by cell count. Error bars indicate standard error of the mean.
(TIF)

Figure S2 (A) Tail suspension test of 16-week old *Xpg^−/−^* and wild type (*wt*) mice showing normal spreading of the hind limbs in *wt* mice, while *Xpg^−/−^* mice display clasping. (B) Rotarod performance of 14-week old *Xpg^−/−^* and *wt* mice. Trials were performed with 1 h intervals; n = 4 animals/group. (C) Average grip strength of the forelimbs and all limbs of 14-week old *Xpg^−/−^* and *wt* mice; n = 4 animals/group. Error bars indicate standard error of the mean. **p<0.01.
(TIF)

Figure S3 (A) Absolute weight of liver, brain, kidney and spleen from 4- and 14-week old *Xpg^−/−^* and wild type (*wt*) males: n = 3 (4 weeks), n = 18 (14 weeks). (B) Representative images of HE stained skin patches of 4-week old *Xpg^−/−^* and *wt* mice. Dotted arrows indicate dermis (d) thickness, which is similar between *wt* and *Xpg^−/−^* mice, while subcutaneous fat (sf) is severely reduced in *Xpg^−/−^* mice as indicated with solid arrows. (C) Relative food intake of *Xpg^−/−^* males (grey triangles), *Xpg^−/−^* females (grey circles), *wt* males (black triangles) and *wt* females (black circles); n = 6 animals/group. Scale bars: 50 µm (B). Error bars indicate standard error of the mean. *p<0.05, **p<0.01.
(TIF)

Figure S4 (A) Representative images of GFAP immunostained sagittal brain sections of 4- and 14-week old *Xpg^−/−^* and wild type (*wt*) mice showing progressive astrocytosis in *Xpg^−/−^* mice. Magnifications of the areas marked with black dotted squares are shown in figure 4A. (B) Confocal images showing a double labeling of Iba1-GFAP in the neocortex of 4- and 14-week old *Xpg^−/−^* and *wt* mice. Right panels are magnifications of the areas marked with the white dotted boxes. Arrowheads indicate resting microglia, while arrows indicate active microglia frequently found in 14-week old *Xpg^−/−^* mice. (C) Confocal images showing a double labeling of Iba1-GFAP in the spinal cord of 14-week old *Xpg^−/−^* and *wt* mice. Right panels are magnifications of the areas marked with the white dotted boxes. Arrowheads indicate resting microglia, while arrows indicate active microglia frequently found in *Xpg^−/−^* mice. (D) Confocal images of double labeled p53-NeuN and p53-S100ß cells in the neocortex of 14-week old *Xpg^−/−^* mice showing p53 staining in both neurons and astrocytes. (E) Confocal images of double labeled p53-NeuN cells in the spinal cord of 14-week old *Xpg^−/−^* mice showing p53 staining mostly in non-neuronal cells. Right panels are magnifications of the areas marked with white dotted boxes. Arrowheads indicate non-neuronal p53 positive cells, while the white arrow indicates a p53 positive neuronal cell. The double arrow points to a p53 positive motor neuron. (F) Confocal images showing a triple immunostaining of GM130, p53 and ChAT in the spinal cord of 14-week old *Xpg^−/−^* mice. Abnormal cis-Golgi in ChAT positive motor neurons can be found in both p53 positive and negative cells. The yellow arrow indicates a p53 positive motor neuron with abnormal cis-Golgi, while the blue arrow points to a p53 negative motor neuron. Scale bars: 1000 µm (A), 200 µm (B, C, E), 20 µm (D, F).
(TIF)

Figure S5 (A) Absolute weight of liver, brain, kidney and spleen from 26- and 52-week old *Alb-Xpg* and wild type (*wt*) males: n = 3 (26 weeks), n = 5 (52 weeks). (B) Albumin concentration in plasma of 26- and 52-week old *Alb-Xpg* and *wt* mice; n≥2 animals/group. (C) Average basal blood glucose levels of 26- and 52-week old *Alb-Xpg* and *wt* mice; n = 3 (26 weeks), n = 5 (52 weeks). (D) Relative mRNA expression levels of several antioxidant genes and the DNA damage response gene *p21* in 26-week old *Emx1-Xpg* liver. All values are corrected for *TubG2, Hprt, and Rps9* (Table S1) expression and normalized to *wt* expression levels; n = 3 animals/group. (E) Relative expression levels of the somatotrophic genes *Ghr, Igf1r and Igf1* in liver tissue of 26-week old *Emx1-Xpg* mice. All values are corrected for *TubG2, Hprt, and Rps9* and normalized to the 26-week *wt* expression levels; n = 3 animals/group. Error bars indicate standard error of the mean. *p<0.05.
(TIF)

Figure S6 (A) Absolute weight of liver, brain, kidney and spleen from 26- and 52-week old *Emx1-Xpg* and wild type (*wt*) females: n = 3 (26 weeks), n = 6 (52 weeks). (B) Average basal blood glucose levels of 26- and 52-week old *Emx1-Xpg* and *wt* mice; n = 3 (26 weeks), n = 6 (52 weeks). (C) Rotarod performance of 26-week old *Emx1-Xpg* and *wt* mice. Average of two trials given for four consecutive days; n = 5 animals/group. (D) Representative images of Mac2 immunostained sagittal brain sections of 26- and 52-week old *Emx1-Xpg* and *wt* mice showing Mac2-positive microgliosis and a progressive decrease in size of the cerebral cortex and hippocampus of *Emx1-Xpg* mice. A thionin counterstaining was used. (E) Magnification of the marked areas indicated in S6D. (F) Representative images of Hsp25 immunostained sagittal brain sections of 26- and 52-week old *Emx1-Xpg* and *wt* mice showing high levels of Hsp25 in the corpus callossum and fimbria fornix of *Emx1-Xpg* mice (arrows), but not in the descending corticofugal axons of the capsula interna which run through the striatum (arrowhead). NCx: neocortex, Str: striatum, Hip: hippocampus, Th: thalamus, Mes: mesencephalon, MeO: medulla oblongata, Cb: cerebellum, cc: corpus callosum, ff: fimbria fornix. Scale bars: 1000 µm (D), 50 µm (E), 500 µm (F). Error bars indicate standard error of the mean. *p<0.05, **p<0.01.
(TIF)

Figure S7 (A) Representative images of GFAP immunostained neocortex sections of 3-week old C67Bl6 *Xpg^−/−^* and *wt* mice showing mild astrocytosis in the *Xpg^−/−^* mice. cc:corpus callossum. (B) Representative images of calbindin immunostained cerebellum sections of 3-week old C67Bl6 *Xpg^−/−^* and *wt* mice showing subtle neuropathology in the *Xpg^−/−^* mice. ml: molecular layer, gl: granular layer. (C) Quantification of p53-positive cells per mm^2^ in neocortex (NCx) and cerebellum (Cb) of 3-week old C67Bl6 *Xpg^−/−^* and *wt* mice. Values are the average of three sections per genotype. Scale bars: 250 µm (A), 100 µm (B, C). Error bars indicate standard error of the mean. **p<0.01.
(TIF)

Table S1 Primer sequences for real-time PCR.
(DOCX)
