# Supplementary figures and images for: Cell-Autonomous Progeroid Changes in Conditional Mouse Models for Repair Endonuclease XPG Deficiency
Source: PLoS Genet. 2014 Oct 9;10(10):e1004686. doi: 10.1371/journal.pgen.1004686 (PMC4191938; doi:10.1371/journal.pgen.1004686)

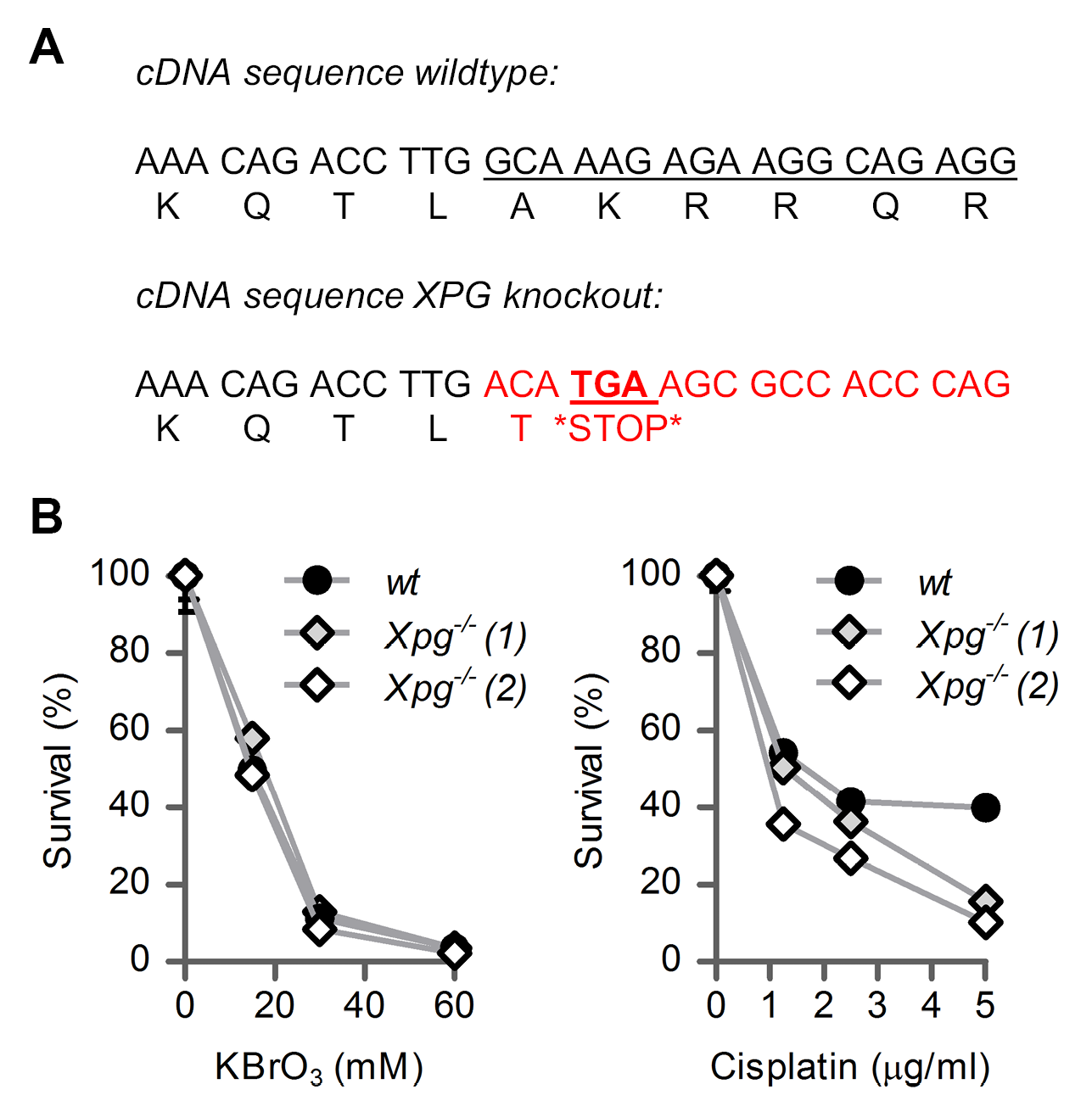

Supplement: Figure S1 — (A) Recombination of Frt and LoxP sites yield a Xpg−/− genetic status with a premature STOP codon in exon 3 as shown by Sänger sequencing of mRNA isolated from Xpg−/− liver tissue. (B) Primary Xpg−/− and wild type (wt) MDFs, cultured at low (3%) O2 levels were treated with the indicated doses of KBrO3 (left) or cisplatin (right) for 1 h. After 48 h recovery, survival was assessed by cell count. Error bars indicate standard error of the mean. (TIF) [file pgen.1004686.s001.tif]

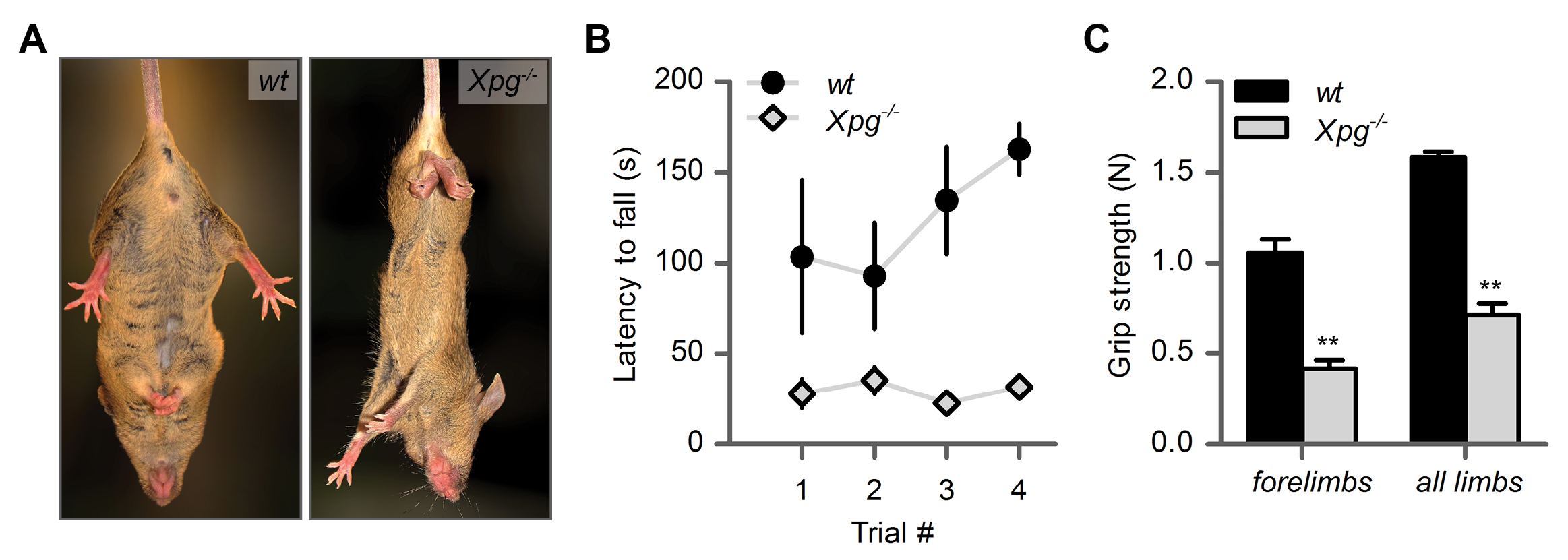

Supplement: Figure S2 — (A) Tail suspension test of 16-week old Xpg−/− and wild type (wt) mice showing normal spreading of the hind limbs in wt mice, while Xpg−/− mice display clasping. (B) Rotarod performance of 14-week old Xpg−/− and wt mice. Trials were performed with 1 h intervals; n = 4 animals/group. (C) Average grip strength of the forelimbs and all limbs of 14-week old Xpg−/− and wt mice; n = 4 animals/group. Error bars indicate standard error of the mean. **p<0.01. (TIF) [file pgen.1004686.s002.tif]

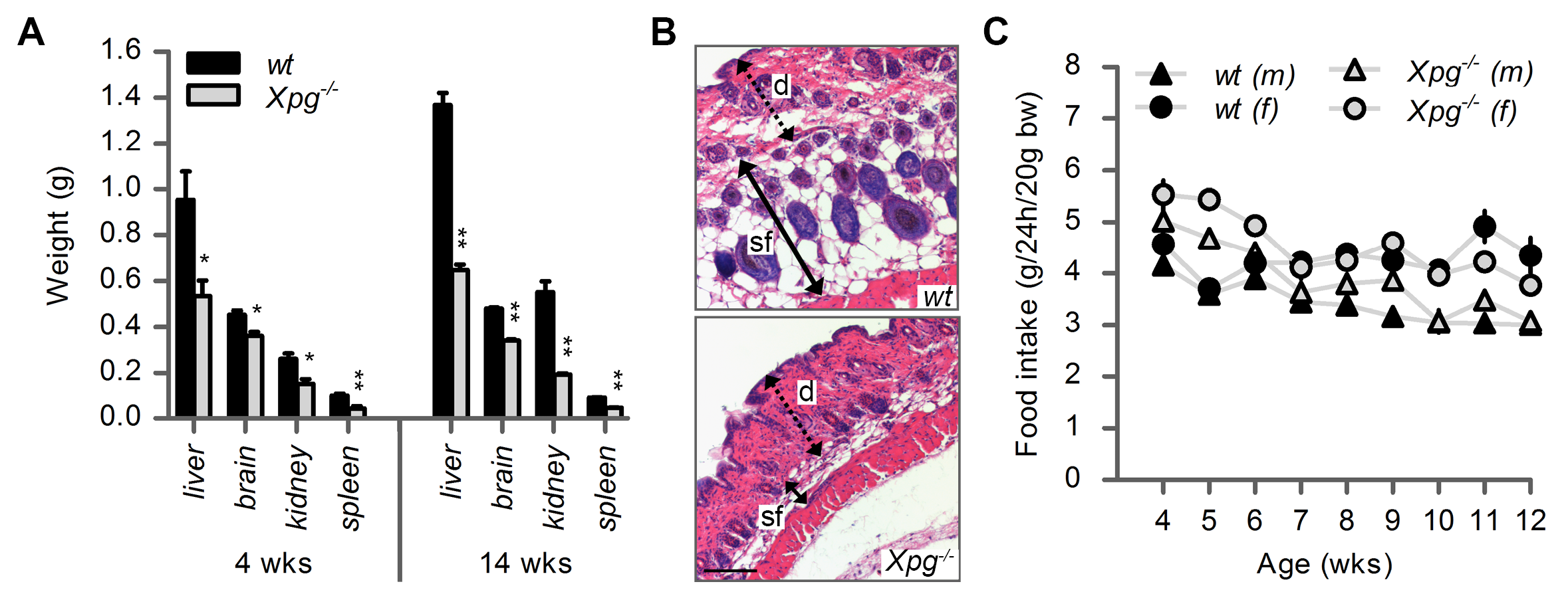

Supplement: Figure S3 — (A) Absolute weight of liver, brain, kidney and spleen from 4- and 14-week old Xpg−/− and wild type (wt) males: n = 3 (4 weeks), n = 18 (14 weeks). (B) Representative images of HE stained skin patches of 4-week old Xpg−/− and wt mice. Dotted arrows indicate dermis (d) thickness, which is similar between wt and Xpg−/− mice, while subcutaneous fat (sf) is severely reduced in Xpg−/− mice as indicated with solid arrows. (C) Relative food intake of Xpg−/− males (grey triangles), Xpg−/− females (grey circles), wt males (black triangles) and wt females (black circles); n = 6 animals/group. Scale bars: 50 µm (B). Error bars indicate standard error of the mean. *p<0.05, **p<0.01. (TIF) [file pgen.1004686.s003.tif]

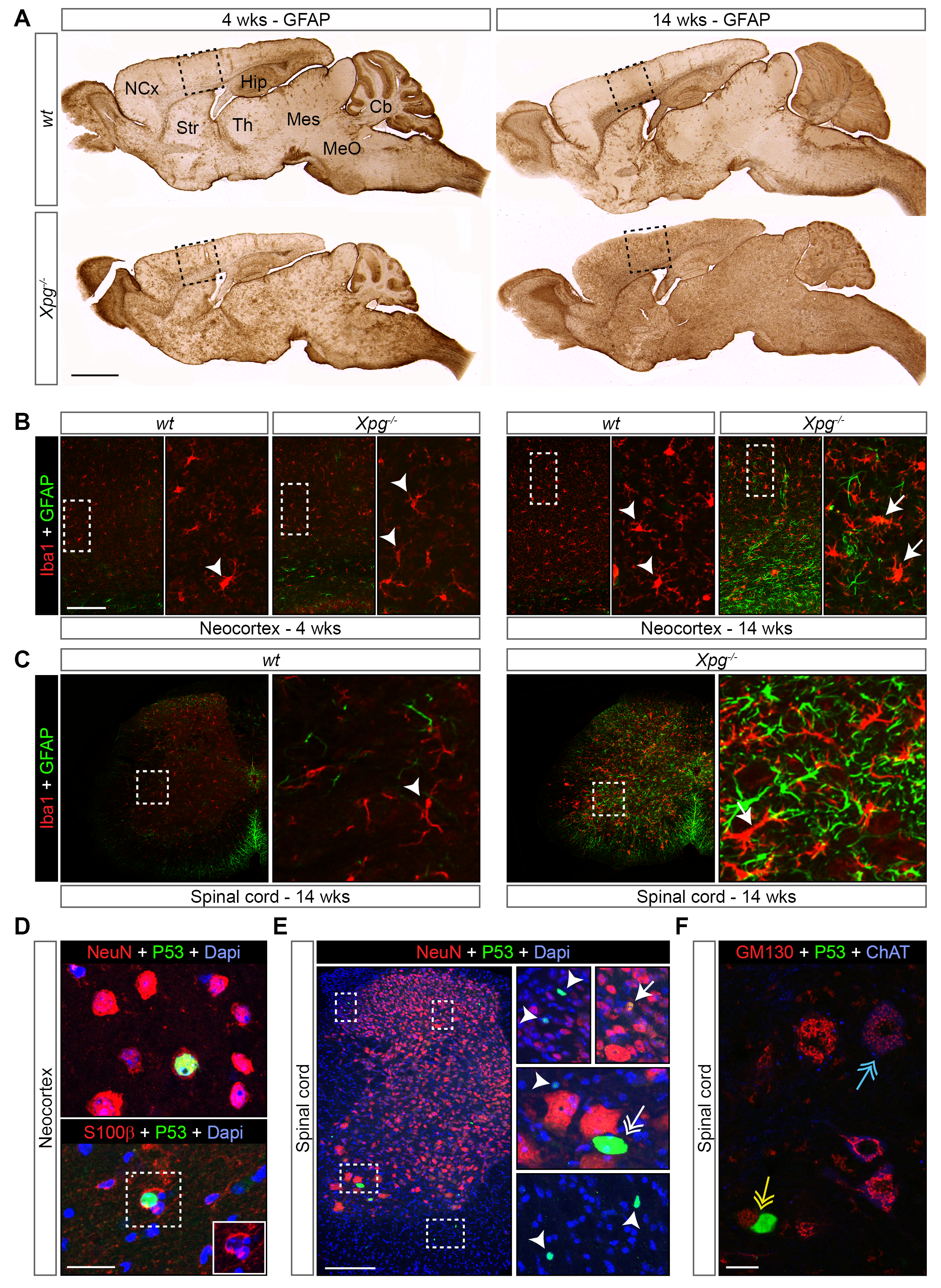

Supplement: Figure S4 — (A) Representative images of GFAP immunostained sagittal brain sections of 4- and 14-week old Xpg−/− and wild type (wt) mice showing progressive astrocytosis in Xpg−/− mice. Magnifications of the areas marked with black dotted squares are shown in figure 4A. (B) Confocal images showing a double labeling of Iba1-GFAP in the neocortex of 4- and 14-week old Xpg−/− and wt mice. Right panels are magnifications of the areas marked with the white dotted boxes. Arrowheads indicate resting microglia, while arrows indicate active microglia frequently found in 14-week old Xpg−/− mice. (C) Confocal images showing a double labeling of Iba1-GFAP in the spinal cord of 14-week old Xpg−/− and wt mice. Right panels are magnifications of the areas marked with the white dotted boxes. Arrowheads indicate resting microglia, while arrows indicate active microglia frequently found in Xpg−/− mice. (D) Confocal images of double labeled p53-NeuN and p53-S100ß cells in the neocortex of 14-week old Xpg−/− mice showing p53 staining in both neurons and astrocytes. (E) Confocal images of double labeled p53-NeuN cells in the spinal cord of 14-week old Xpg−/− mice showing p53 staining mostly in non-neuronal cells. Right panels are magnifications of the areas marked with white dotted boxes. Arrowheads indicate non-neuronal p53 positive cells, while the white arrow indicates a p53 positive neuronal cell. The double arrow points to a p53 positive motor neuron. (F) Confocal images showing a triple immunostaining of GM130, p53 and ChAT in the spinal cord of 14-week old Xpg−/− mice. Abnormal cis-Golgi in ChAT positive motor neurons can be found in both p53 positive and negative cells. The yellow arrow indicates a p53 positive motor neuron with abnormal cis-Golgi, while the blue arrow points to a p53 negative motor neuron. Scale bars: 1000 µm (A), 200 µm (B, C, E), 20 µm (D, F). (TIF) [file pgen.1004686.s004.tif]

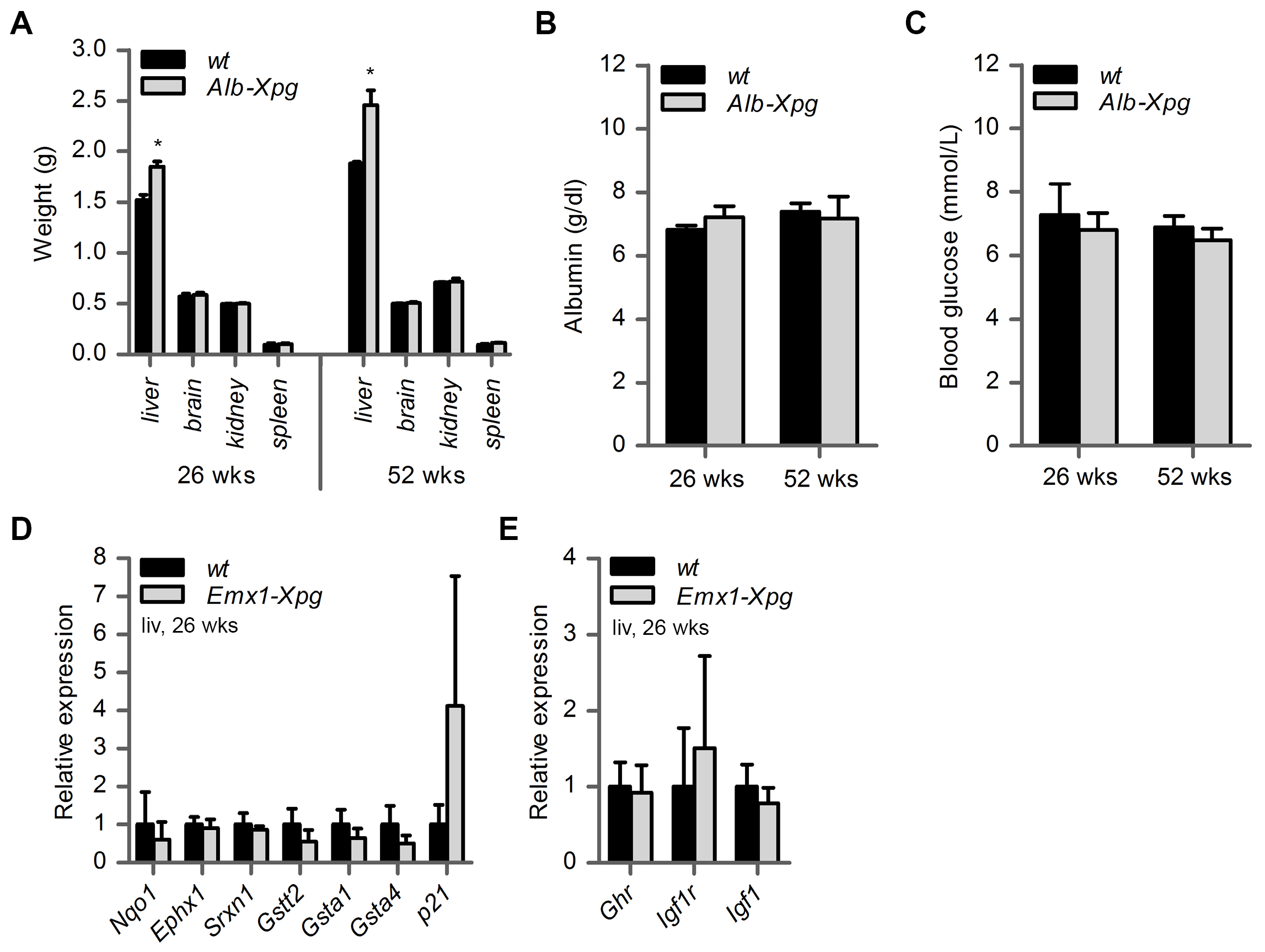

Supplement: Figure S5 — (A) Absolute weight of liver, brain, kidney and spleen from 26- and 52-week old Alb-Xpg and wild type (wt) males: n = 3 (26 weeks), n = 5 (52 weeks). (B) Albumin concentration in plasma of 26- and 52-week old Alb-Xpg and wt mice; n≥2 animals/group. (C) Average basal blood glucose levels of 26- and 52-week old Alb-Xpg and wt mice; n = 3 (26 weeks), n = 5 (52 weeks). (D) Relative mRNA expression levels of several antioxidant genes and the DNA damage response gene p21 in 26-week old Emx1-Xpg liver. All values are corrected for TubG2, Hprt, and Rps9 (Table S1) expression and normalized to wt expression levels; n = 3 animals/group. (E) Relative expression levels of the somatotrophic genes Ghr, Igf1r and Igf1 in liver tissue of 26-week old Emx1-Xpg mice. All values are corrected for TubG2, Hprt, and Rps9 and normalized to the 26-week wt expression levels; n = 3 animals/group. Error bars indicate standard error of the mean. *p<0.05. (TIF) [file pgen.1004686.s005.tif]

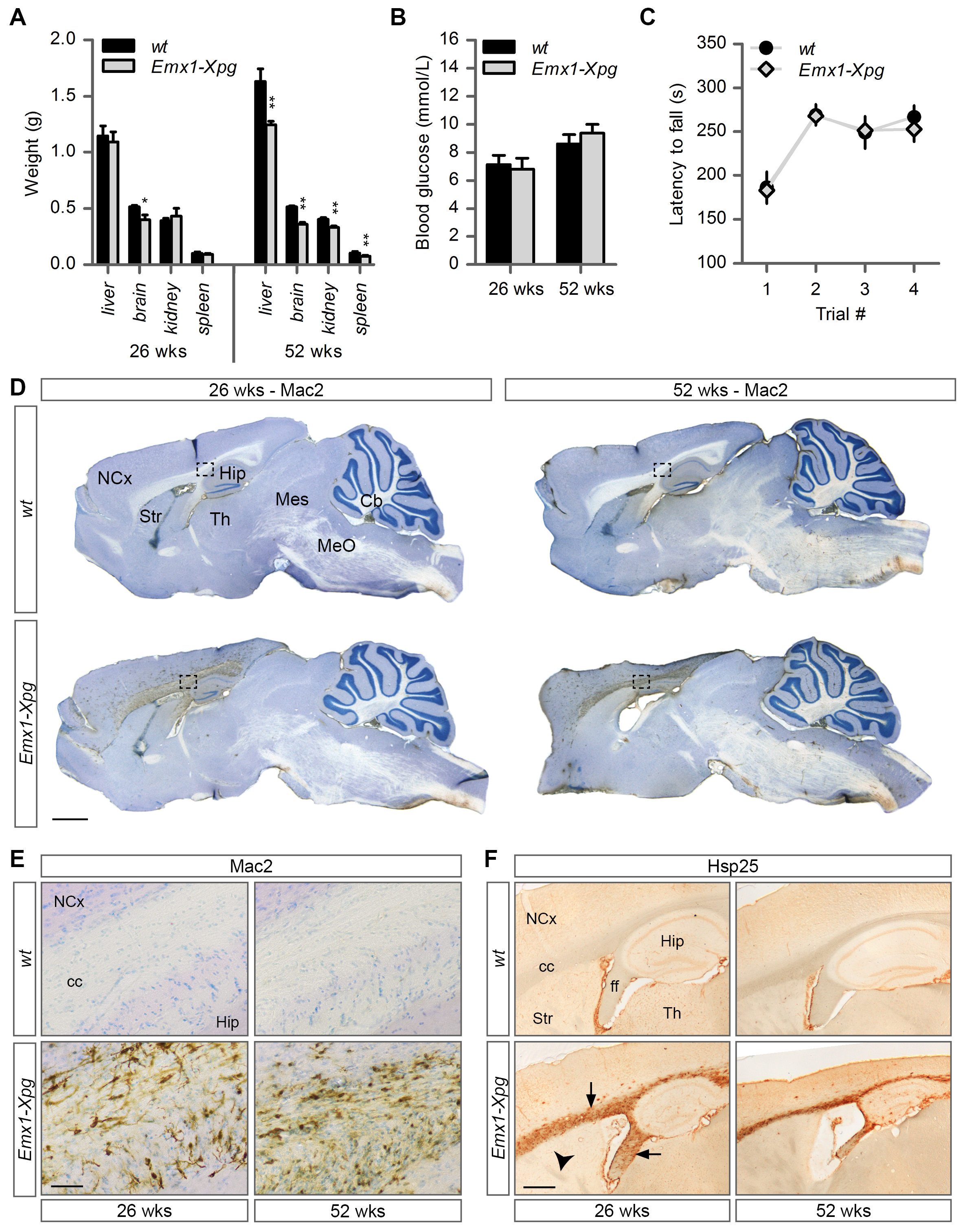

Supplement: Figure S6 — (A) Absolute weight of liver, brain, kidney and spleen from 26- and 52-week old Emx1-Xpg and wild type (wt) females: n = 3 (26 weeks), n = 6 (52 weeks). (B) Average basal blood glucose levels of 26- and 52-week old Emx1-Xpg and wt mice; n = 3 (26 weeks), n = 6 (52 weeks). (C) Rotarod performance of 26-week old Emx1-Xpg and wt mice. Average of two trials given for four consecutive days; n = 5 animals/group. (D) Representative images of Mac2 immunostained sagittal brain sections of 26- and 52-week old Emx1-Xpg and wt mice showing Mac2-positive microgliosis and a progressive decrease in size of the cerebral cortex and hippocampus of Emx1-Xpg mice. A thionin counterstaining was used. (E) Magnification of the marked areas indicated in S6D. (F) Representative images of Hsp25 immunostained sagittal brain sections of 26- and 52-week old Emx1-Xpg and wt mice showing high levels of Hsp25 in the corpus callossum and fimbria fornix of Emx1-Xpg mice (arrows), but not in the descending corticofugal axons of the capsula interna which run through the striatum (arrowhead). NCx: neocortex, Str: striatum, Hip: hippocampus, Th: thalamus, Mes: mesencephalon, MeO: medulla oblongata, Cb: cerebellum, cc: corpus callosum, ff: fimbria fornix. Scale bars: 1000 µm (D), 50 µm (E), 500 µm (F). Error bars indicate standard error of the mean. *p<0.05, **p<0.01. (TIF) [file pgen.1004686.s006.tif]

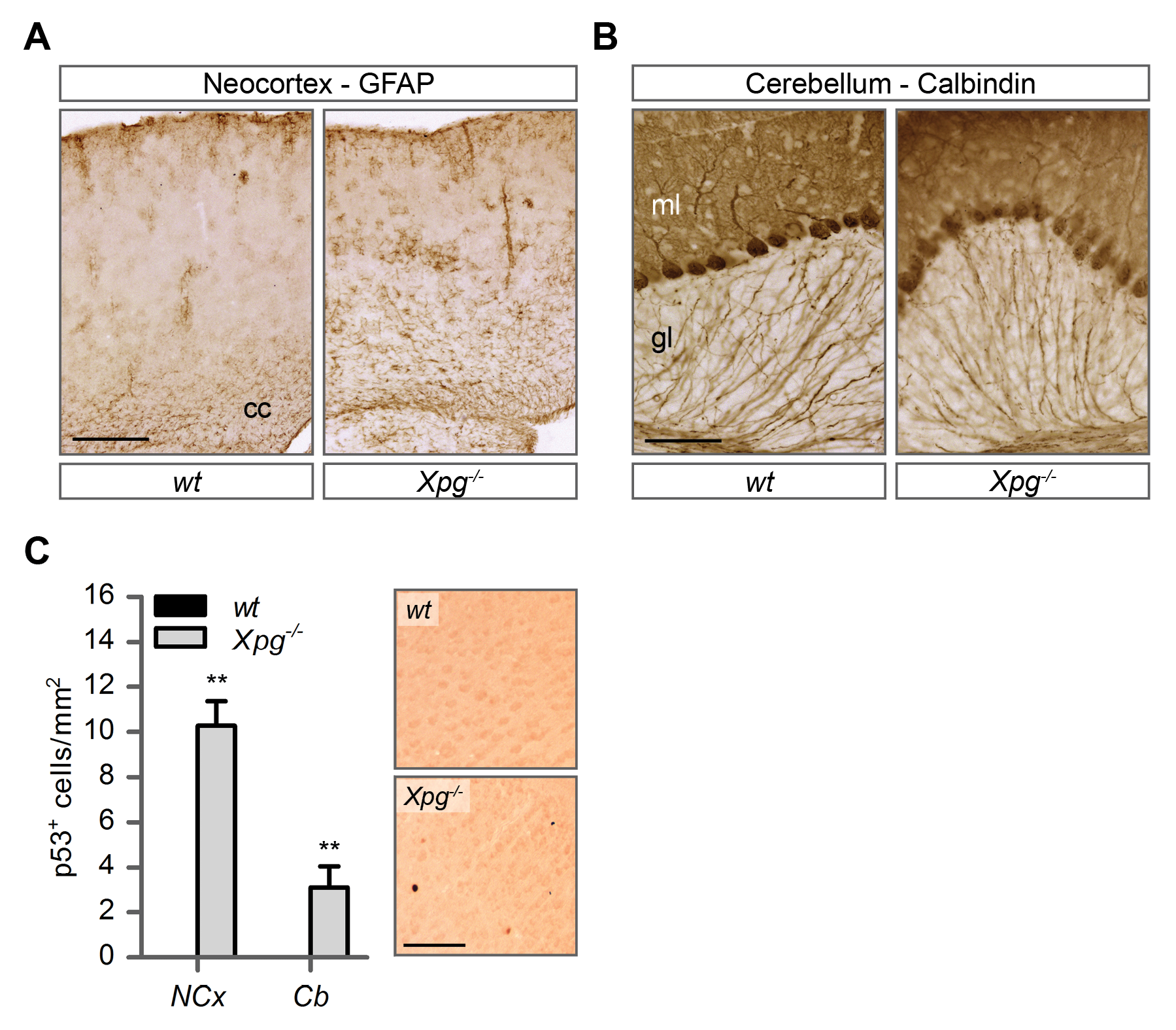

Supplement: Figure S7 — (A) Representative images of GFAP immunostained neocortex sections of 3-week old C67Bl6 Xpg−/− and wt mice showing mild astrocytosis in the Xpg−/− mice. cc:corpus callossum. (B) Representative images of calbindin immunostained cerebellum sections of 3-week old C67Bl6 Xpg−/− and wt mice showing subtle neuropathology in the Xpg−/− mice. ml: molecular layer, gl: granular layer. (C) Quantification of p53-positive cells per mm2 in neocortex (NCx) and cerebellum (Cb) of 3-week old C67Bl6 Xpg−/− and wt mice. Values are the average of three sections per genotype. Scale bars: 250 µm (A), 100 µm (B, C). Error bars indicate standard error of the mean. **p<0.01. (TIF) [file pgen.1004686.s007.tif]
