# Supplementary material for: Cell-Autonomous Progeroid Changes in Conditional Mouse Models for Repair Endonuclease XPG Deficiency
Source: PLoS Genet. 2014 Oct 9;10(10):e1004686. doi: 10.1371/journal.pgen.1004686 (PMC4191938; doi:10.1371/journal.pgen.1004686)
Supplement: Table S1 — Primer sequences for real-time PCR. (DOCX) [file pgen.1004686.s008.docx]

| **Gene** | **Forward primer (5’ to 3’)** | **Reverse primer (5’ to 3’)** |
| --- | --- | --- |
| ***Nqo1*** | GGTAGCGGCTCCATGTACTC | GAGTGTGGCCAATGCTGTAA |
| ***Ephx1*** | GAGTGGAGGAACTGCACACC | AGCACAGAAGCCAGGATGA |
| ***Srxn1*** | TGAGCAGCTCCTCTGATGTG | GCTGAGGTGACAATTGACTATGG |
| ***Gstt2*** | CGAGCAATTCTCCCAGGTGA | TATTCGTGGACTTGGGCACG |
| ***Gsta1*** | CTTCTGACCCCTTTCCCTCT | ATCCATGGGAGGCTTTCTCT |
| ***Gsta4*** | TCGATGGGATGATGCTGAC | CATCTGCATACATGTCAATCCTG |
| ***Ho1*** | CAGGTGATGCTGACAGAGGA | ATGGCATAAATTCCCACTGC |
| ***Nrf2*** | AGGACATGGAGCAAGTTTGG | TCTGTCAGTGTGGCTTCTGG |
| ***p21*** | CAAGAGGCCCAGTACTTCCT | CAATCTGCGCTTGGAGTGAT |
| ***Ghr*** | ATTCACCAAGTGTCGTTCCC | TCCATTCCTGGGTCCATTCA |
| ***Igf1r*** | ACGACAACACAACCTGCGT | AACGAAGCCATCCGAGTCA |
| ***Igf1*** | TGCTTGCTCACCTTCACCA | CAACACTCATCCACAATGCC |
| ***TubG2*** | CAGACCAACCACTGCTACAT | AGGGAATGAAGTTGGCCAGT |
| ***Hprt*** | TGATAGATCCATTCCTATGACTGTAGA | AAGACATTCTTTCCAGTTAAAGTTGAG |
| ***Rps9*** | ATCCGCCAACGTCACATTA | TCTTCACTCGGCCTGGAC |
